# Histone proteomics reveals novel post-translational modifications in breast cancer

**DOI:** 10.18632/aging.102577

**Published:** 2019-12-08

**Authors:** Angela Mena Perri, Valter Agosti, Erika Olivo, Antonio Concolino, MariaTeresa De Angelis, Laura Tammè, Claudia Vincenza Fiumara, Giovanni Cuda, Domenica Scumaci

**Affiliations:** 1 Laboratory of Proteomics, Research Center on Advanced Biochemistry and Molecular Biology, Department of Experimental and Clinical Medicine, Magna Græcia University of Catanzaro, Salvatore Venuta University Campus, Catanzaro, Italy; 2 Laboratory of Molecular Oncology, Department of Experimental and Clinical Medicine, Magna Graecia University, Catanzaro, Italy, CIS for Genomics and Molecular Pathology, Magna Graecia University, Catanzaro, Italy; 3 Stem Cell Laboratory, Research Center of Advanced Biochemistry and Molecular Biology, Department of Experimental and Clinical Medicine University “Magna Graecia” of Catanzaro, Salvatore Venuta University Campus, Catanzaro, Italy

**Keywords:** breast cancer, histone, 2D TAU gel, phosphorylation, FAK

## Abstract

Histones and their variants are subjected to several post-translational modifications (PTMs). Histones PTMs play an important role in the regulation of gene expression and are critical for the development and progression of many types of cancer, including breast cancer. In this study, we used two-dimensional TAU/SDS electrophoresis, coupled with mass spectrometry for a comprehensive profiling of histone PTMs in breast cancer cell lines.

Proteomic approach allowed us to identify 85 histone PTMs, seventeen of which are not reported in the UniProt database. Western blot analysis was performed to confirm a peculiar pattern of PTMs in the sporadic and hereditary breast cancer cell lines compared to normal cells. Overlapping mass spectrometry data with western blotting results, we identified, for the first time to our knowledge, a tyrosine phosphorylation on histone H1, which is significantly higher in breast cancer cells. Additionally, by inhibiting specific signaling paths, such as PI3K, PPARγ and FAK pathways, we established a correlation between their regulation and the presence of new histone PTMs. Our results may provide new insight on the possible implication of these modifications in breast cancer and may offer new perspectives for future clinical applications.

## INTRODUCTION

In eukaryotic cells, DNA is packaged into chromatin. The level of compaction derives from the degree of DNA winding around nucleosomes [1]. Each nucleosome consists of 147bp of DNA wrapped around an octamer core containing one histone H3–H4 tetramer and two histone H2A-H2B dimers. Histones are very basic proteins highly conserved throughout evolution, organized in three domains: the hydrophobic central globular domain that interacts with DNA, and the very basic unstructured C- and N-terminal tails [2, 3]. Tails protrude away from the nucleosome and are frequently subjected to post-translational modifications (PTMs). In addition to histones of the core particle, the linker histone H1 binds the DNA on the surface of the nucleosomal core and completes the nucleosome. The histone linker is required to stabilize the highly ordered chromatin structure [4]. The action of H1 histone influences the nucleosomal repeat length (NRL), thereby modulating the accessibility of chromatin for transcription [2, 4].

The degree of packaging of chromatin is highly influenced by numerous factors, including histone PTMs [1]. Enzymes called *writers* and *erasers* add and remove PTMs from histones, affecting inter/intra-nucleosomal interactions and their binding to DNA. In addition, histone readers specifically bind certain PTMs, resulting in specific responses at the level of transcription, DNA repair and replication [3].

Histones and histone-variants represent a key class of proteins able to trigger the encoding of epigenetic information as well as the regulation of gene expression [5]. Histone PTMs profiles (histone code) are known to be altered in many types of cancer, including breast cancer, the most frequent neoplasia among women. Specific histone PTMs are associated with breast cancer development and prognosis, such as H3K9ac, H3K9me2-3, H4K16ac and H4K20me3 [3, 6–10]. A plethora of studies suggests a pivotal role of histone modifications in the onset as well as in the progression of breast cancer. Therefore, profiling and characterization of histone isoforms and their PTMs may contribute to unravel the molecular mechanisms underlying breast tumorigenesis. Moreover, the role of epigenetics in sporadic as well as in hereditary breast cancer needs to be deepened in order to provide novel targets for the development of personalized therapeutic approaches.

In this work, we applied 2D-TAU/SDS gel electrophoresis coupled to LC-MS/MS analysis to identify and characterize histone PTMs profiles in normal mammary epithelial cell line MCF10 and in two distinct breast cancer cell lines: MCF7 (sporadic breastcancer model) and HCC1937 (BRCA1^-/-^ hereditary breast cancer model) [11–14].

Seventeen novel histone marks were identified. In addition, 2D-TAU Western blot analysis was applied to differentially profile the tyrosine phosphorylation pattern in all cell lines

The most striking result is the identification of a tyrosine phosphorylation on the histone H1, that increases in breast cancer cells and correlates with the proliferative status. To the best of our knowledge, this is the first report of such a finding.

Ultimately, we identify additional putative cancer-related histone marks, we reveal quantitative differences of PTMs in different cellular models of breast cancer and, suggesting a pivotal role of these modifications in proliferation, we provide a substantial input for further investigations.

## RESULTS

### 2D TAU gel of histone PTMs in breast cell lines

**Figure 1:**
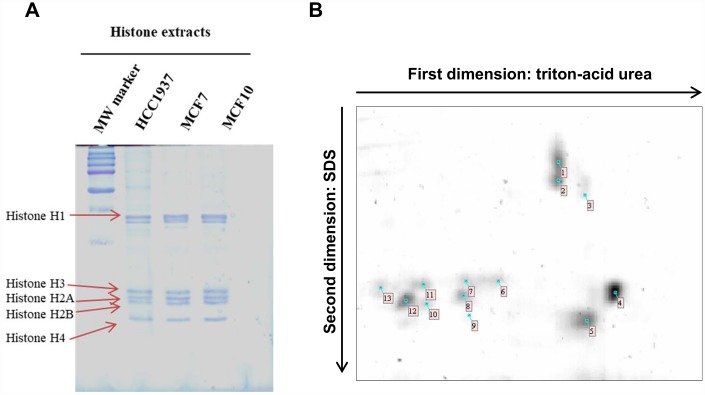
**1D TAU gel and 2D TAU gel map of histones in breast cancer cells.** (Panel **A**) The image shows a peculiar separation pattern of histone isoforms, extract from HCC1937, MCF7 and MCF10 cells lines, using 1D-TAU gel. (Panel **B**) Representative 2D TAU PAGE of histones extract from MCF7 cells. Histones were first resolved by TAU gel and subsequently separated using SDS gel. Spots extracted and analyzed by mass spectrometry are noted on the gel map. All experiments were repeated three times using biologic replicates. Numbered spots are described on table 1 where for each spot is reported the id number, the accession number, histone description, the number of identified peptides, the percentage of sequence coverage, molecular weight and isoelectric point.

Histones were isolated from mammalian cell lines and proteins content was determined using Bradford Protein Assay (Bio-Rad) according to the manufacturer’s instructions with human serum albumin (Sigma Aldrich) as standard. Twenty μg of each sample were loaded on a 1D TAU-gel to assess the efficiency of the isolation methods. The gel, relative to the separation of histones is shown in Figure 1A. As expected, the separation pattern of histone isoforms was found coherent with previous literature [11].

Two-dimensional (2D) TAU gel allowed us to resolve each histone isoform. Gel maps are shown as Figure 1B. By means of this approach, we obtained a map of thirteen protein spots. Images analysis, performed using image master 2d platinum software, allows us to focus specifically on the histone isoforms differentially expressed in cancer cells compared to normal cells. Differentially expressed histone spots are marked with a progressive number on the 2D gel map.

### Mass spectrometry analysis of TAU gel spot reveals novel PTMs in breast cancer cells

**Figure 2:**
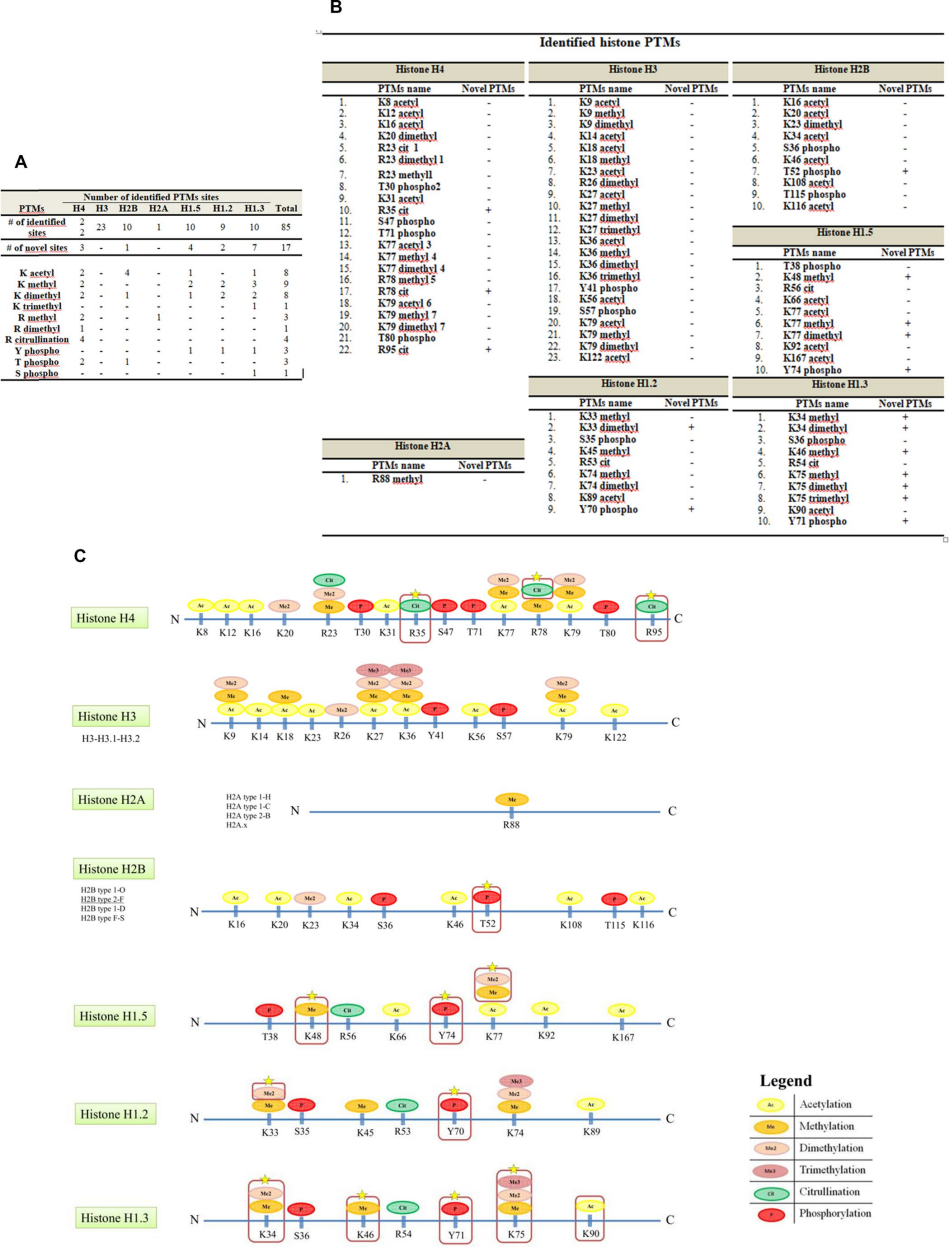
**Histone PTMs sites identified by this study.** (**A**) The table summarizes all the PTMs sites identified. Using the described approach, we identified a total of eighty-five histones PTMs, seventeen of these were not previously described on the UniProt database. (**B**) The identified novel modifications consist of 5 lysine methylation, 4 lysine dimethylation, 1 lysine trimethylation, 3 arginine citrullination, 1 threonine phosphorylation and 3 tyrosine phosphorylation. Mass spectrometry data are averages of three biologic replicates. (**C**) Novel and known sites of PTM along histones sequences. The identified modifications consist of acetylation, methylation, dimethylation, trimethylation, citrullination and phosphorylation. Red boxes indicate novel modifications.

Gel spots were in-gel digested with trypsin and analyzed by mass spectrometry. Table 1 summarizes the results of the LC-MS/MS identifications. For each identification, we reported accession number, number of identified peptides and percentage of sequence coverage. As expected, each protein spot corresponded to a specific histone isoform. As shown in Figure 2, we identified several modifications on all the five canonical histones. Among these, some had been previously reported in the UniProt database (http://www.uniprot.org), many others were novel. PTMs we found consist of lysine acetylation, lysine and arginine methylation, dimethylation, trimethylation, arginine citrullination and threonine, tyrosine and serine phosphorylation.

**Table 1:** LC-MS/MS identifications.

**Spot id**	**Accession number**	**Description**	**Coverage**	**Unique Peptides**	**Score**	**MW [kDa]**	**calc. pI**
**Spot 1**	P16401	Histone H1.5 OS=Homo sapiens GN=HIST1H1B PE=1 SV=3 - [H15_HUMAN]	35,84	12	237,42	22,6	10,92
**Spot 2**	P16403	Histone H1.2 OS=Homo sapiens GN=HIST1H1C PE=1 SV=2 - [H12_HUMAN]	34,74	12	390,76	21,4	10,93
	P16402	Histone H1.3 OS=Homo sapiens GN=HIST1H1D PE=1 SV=2 - [H13_HUMAN]	35,29	11	229,94	22,3	11,02
**Spot 3**	P07305	Histone H1.0 OS=Homo sapiens GN=H1F0 PE=1 SV=3 - [H10_HUMAN]	50,00	13	92,32	20,9	10,84
	P07305-2	Isoform 2 of Histone H1.0 OS=Homo sapiens GN=H1F0 - [H10_HUMAN]	33,33	8	155,68	19,2	10,83
**Spot 4**	Q5QNW6	Histone H2B type 2-F OS=Homo sapiens GN=HIST2H2BF PE=1 SV=3 - [H2B2F_HUMAN]	80,95	6	719,93	13,9	10,32
	P06899	Histone H2B type 1-J OS=Homo sapiens GN=HIST1H2BJ PE=1 SV=3 - [H2B1J_HUMAN]	79,37	3	691,82	13,9	10,32
	O60814	Histone H2B type 1-K OS=Homo sapiens GN=HIST1H2BK PE=1 SV=3 - [H2B1K_HUMAN]	41,27	6	30,84	13,9	10,32
	P58876	Histone H2B type 1-D OS=Homo sapiens GN=HIST1H2BD PE=1 SV=2 - [H2B1D_HUMAN]	80,95	4	943,16	13,9	10,32
	P23527	Histone H2B type 1-O OS=Homo sapiens GN=HIST1H2BO PE=1 SV=3 - [H2B1O_HUMAN]	80,95	3	630,95	13,9	10,32
	Q99880	Histone H2B type 1-L OS=Homo sapiens GN=HIST1H2BL PE=1 SV=3 - [H2B1L_HUMAN]	73,81	4	255,71	13,9	10,32
	Q8N257	Histone H2B type 3-B OS=Homo sapiens GN=HIST3H2BB PE=1 SV=3 - [H2B3B_HUMAN]	73,81	4	223,44	13,9	10,32
**Spot 5**	P62805	Histone H4 OS=Homo sapiens GN=HIST1H4A PE=1 SV=2 - [H4_HUMAN]	84,47	19	1058,19	11,4	11,36
**Spot 6**	P68431	Histone H3.1 OS=Homo sapiens GN=HIST1H3A PE=1 SV=2 - [H31_HUMAN]	62,5	14	144,31	15,4	11,12
**Spot 7**	Q71DI3	Histone H3.2 OS=Homo sapiens GN=HIST2H3A PE=1 SV=3 - [H32_HUMAN]	83,82	3	380,59	15,4	11,27
	K7EK07	Histone H3 (Fragment) OS=Homo sapiens GN=H3F3B PE=3 SV=1 - [K7EK07_HUMAN]	65,91	4	196,58	14,9	11,30
	P68431	Histone H3.1 OS=Homo sapiens GN=HIST1H3A PE=1 SV=2 - [H31_HUMAN]	63,97	4	333,96	15,4	11,12
**Spot 8**	Q16777	Histone H2A type 2-C OS=Homo sapiens GN=HIST2H2AC PE=1 SV=4 - [H2A2C_HUMAN]	63,57	7	136,64	14,0	10,90
	Q96KK5	Histone H2A type 1-H OS=Homo sapiens GN=HIST1H2AH PE=1 SV=3 - [H2A1H_HUMAN]	58,59	5	108,22	13,9	10,89
**Spot 9**	P0C0S5	Histone H2A.Z OS=Homo sapiens GN=H2AFZ PE=1 SV=2 - [H2AZ_HUMAN]	31,25	2	37,33	13,5	10,58
**Spot 10**	Q96KK5	Histone H2A type 1-H OS=Homo sapiens GN=HIST1H2AH PE=1 SV=3 - [H2A1H_HUMAN]	79,69	3	224,94	13,9	10,89
	Q93077	Histone H2A type 1-C OS=Homo sapiens GN=HIST1H2AC PE=1 SV=3 - [H2A1C_HUMAN]	78,46	2	222,63	14,1	11,05
**Spot 11**	P68431	Histone H3.1 OS=Homo sapiens GN=HIST1H3A PE=1 SV=2 - [H31_HUMAN]	63,97	20	587,61	15,4	11,12
**Spot 12**	Q96KK5	Histone H2A type 1-H OS=Homo sapiens GN=HIST1H2AH PE=1 SV=3 - [H2A1H_HUMAN]	79,69	3	224,94	13,9	10,89
	P16104	Histone H2A.x OS=Homo sapiens GN=H2AFX PE=1 SV=2 - [H2AX_HUMAN]	65,03	2	104,67	15,13	10,74
	P0C0S5	Histone H2A.Z OS=Homo sapiens GN=H2AFZ PE=1 SV=2 - [H2AZ_HUMAN]	56,25	4	116,77	13,5	10,58
	Q93077	Histone H2A type 1-C OS=Homo sapiens GN=HIST1H2AC PE=1 SV=3 - [H2A1C_HUMAN]	78,46	2	222,64	14,1	11,05
	Q8IUE6	Histone H2A type 2-B OS=Homo sapiens GN=HIST2H2AB PE=1 SV=3 - [H2A2B_HUMAN]	60,00	3	75,51	14,0	10,89
**Spot 13**	K7EK07	Histone H3 (Fragment) OS=Homo sapiens GN=H3F3B PE=3 SV=1 - [K7EK07_HUMAN]	63,64	4	183,74	14,9	11,30
	P68431	Histone H3.1 OS=Homo sapiens GN=HIST1H3A PE=1 SV=2 - [H31_HUMAN]	61,76	4	155,95	15,4	11,12
	Q71DI3	Histone H3.2 OS=Homo sapiens GN=HIST2H3A PE=1 SV=3 - [H32_HUMAN]	85,29	5	339,42	15,4	11,27

In the table are summarized the results of the LC-MS/MS identifications. For each identification, we reported the number by which spot are marked on Figure 1, the accession number, the number of identified peptides and the % of sequence coverage. Mass spectrometry data are averages of three biologic replicates.

As summarized in Figure 2A we identified a total of eighty-five histones PTMs, 17 of which not previously described on the UniProt database. Figure 2B–2C lists all histone isoforms PTMs identified in this study. MS/MS data are provided as Supplementary Figure 3.

### Western blot analysis of specific histone PTMs in breast cancer cell lines

Our analytic procedure was validated by analyzing, through western blot experiment, the expression of specific histone PTMs known to be dysregulated in breast cancer.

The availability of commercial antibodies to specific sites allowed us to highlight important differences between the normal mammary epithelial cell line and breast cancer cell lines.

**Figure 3:**
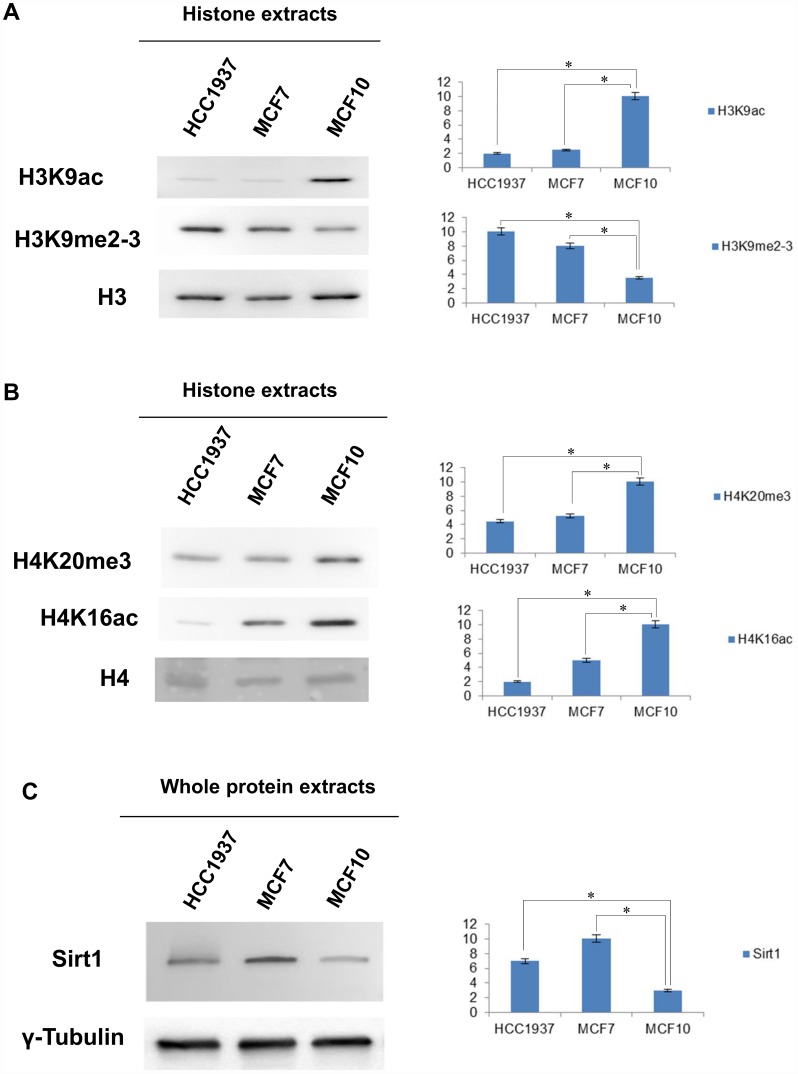
**Western blot and densitometry analysis relative to the expression of histone marks and SIRT1 in MCF10, MCF7 and HCC1937 cells.** (**A**) Western blot and densitometry analysis relative to the expression of histone marks in MCF10, MCF7 and HCC1937 cells. (**A**) H3K9ac, H3K9me2-3, PMTs signal were normalized against the total level of histone H3. (**B**) H4K20me3, H4K16Ac, PMTs signal were normalized against the total level histone H4. (**C**) Western blot and densitometry analysis of the expression levels of SIRT1 in whole protein extracts from MCF10, MCF7 and HCC1937 cells. A goat polyclonal anti-γ-Tubulin antibody was used (C-20) to confirm an equal loading of proteins. The assays were repeated in three independent biological replicates and statistically significant differences were determined using one-way ANOVA followed by Dunnett's multiple comparisons test. Data are expressed as mean ± SEM (N =3), p-value <0.05.

Western blot analysis was done on four histone marks: H4K16ac, H3K9ac, H4K20me3 and H3K9me2-3. Expression of each modification is shown in Figure 3A.

The dysregulation of peculiar histone marks was observed in both cancer cells (MCF7 and HCC1937) compared to normal cells (MCF10).

We found low levels of H4K16ac and H4K20me3, fully in agreement with current literature that links aberrant low levels of these modifications with cell invasiveness, and breast cancer progression [9, 10]. We also detected in our cancer cells high levels of H3K9me2-3, enforcing the notion that the epigenetic silencing of several tumor suppressor genes is a key event in breast cancer cells [15, 17].

As expected, the levels of H3K9ac was low being methylation and acetylation mutually exclusive [17, 18].

A key regulator of H3K9 acetylation is the NAD-dependent histone deacetylase SIRT1 that is overexpressed in many types of cancer including breast cancer. SIRT1 plays an important role in several cellular processes such as chromatin assembly, gene transcription and inflammation. Usually, the enzyme is able to act on two groups of acetylated proteins: histone and non-histone proteins. For histone targets, the status of acetylation/deacetylation determines whether chromatin is accessible for gene transcription. SIRT1 directly deacetylates H1K26, H3K9, and H4K16 and influences DNA compaction, silencing gene transcription [19]. We performed Western blot analysis of total SIRT1 levels in whole protein extracts from MCF10, MCF7 and HCC1937 cells. Consistently, levels of SIRT1 were significantly increased in breast cancer cells compared to normal mammary epithelial cells as shown in Figure 3B.

### Tyrosine phosphorylation profiling of histones by 2D Western Blot analysis

**Figure 4:**
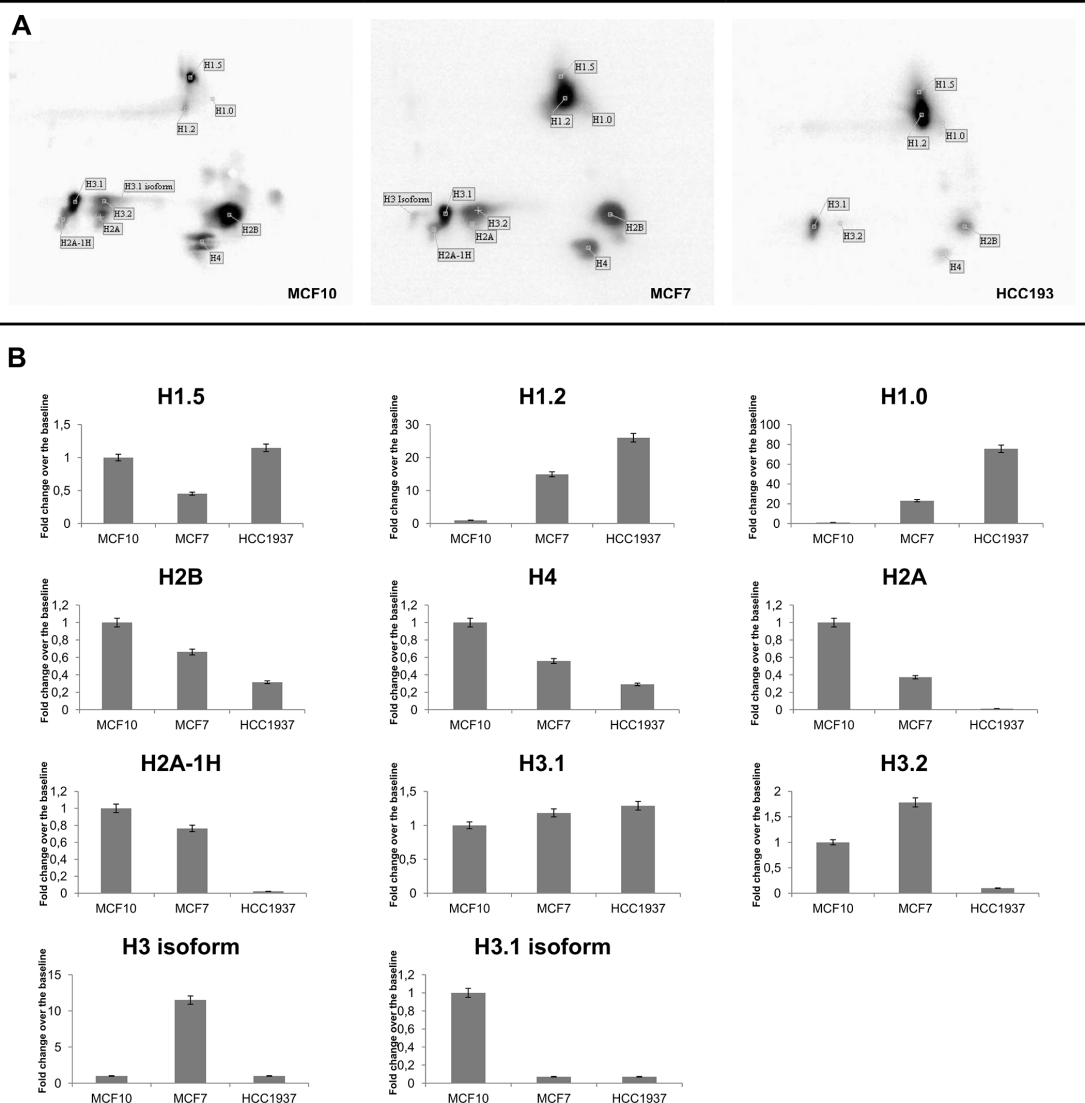
**Tyrosine phosphorylation profiling of histones by 2D Western Blot analysis**. 2D TAU Western blot of histone tyrosine phosphorylation pattern (panel **A**) and densitometry analysis of differentially expressed spots (panel **B**). The assay was repeated in three independent biological replicates; statistical analysis was done by one-way ANOVA, followed by Dunnett's multiple comparisons test. Differences were considered significant when P≤0.05(*). Data are expressed as mean ± SEM (N =3). Images relative to proteins normalization are provided as Supplementary Figure 2.

The overall pattern of histone tyrosine phosphorylation of all three cellular models was analyzed by 2D TAU gel, followed by Western blotting with anti-phospho-Y antibodies. Figure 4 illustrates the tyrosine phosphorylation patterns. In panel A are shown representative images of 2D TAU Western blot, and in the panel B is reported the corresponding densitometry analysis. The most significant finding is the detection of a phosphorylation in tyrosine on the histone H1.

### H1 histone tyrosine phosphorylation results increased in breast cancer cell lines

**Figure 5:**
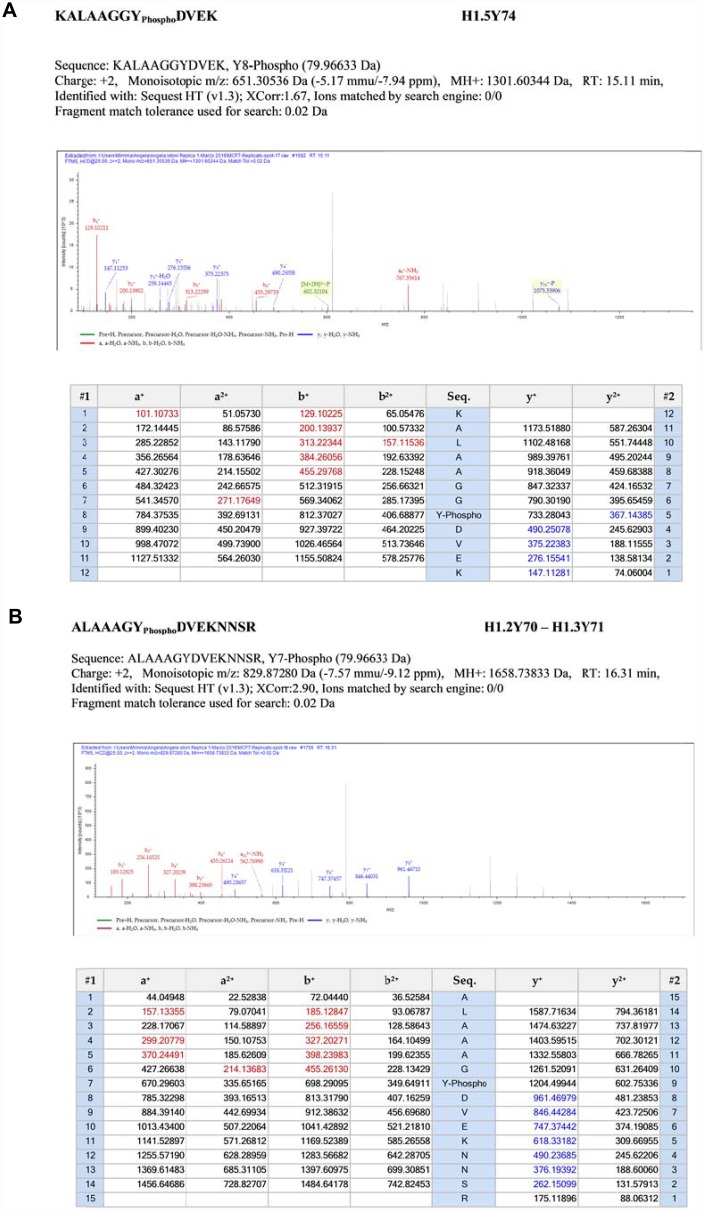
**Fragmentation MS/MS spectra.** (Panel **A**) Fragmentation MS/MS spectra of the modified peptide carrying the novel PTMs. MS/MS data are referred to histone H1.5. Data were analyzed by Proteome Discoverer 1.4 software. MS/MS data were searched on the Human UniProt database. False discovery rate (FDR) of peptide identifications was estimated using the “Target-decoy PSM validator” node in Proteome Discoverer. Cut off filters 95% confidence and a minimum of two peptide identifications per protein. (Panel **B**) Fragmentation MS/MS spectra of the modified peptide carrying the novel PTMs. MS/MS data are referred to histones H1.2 and H1.3. Data were analyzed by Proteome Discoverer 1.4 software. MS/MS data were searched on the Human UniProt database. False discovery rate (FDR) of peptide identifications was estimated using the “Target-decoy PSM validator” node in Proteome Discoverer. Cut off filters 95% confidence and a minimum of two peptide identifications per protein.

Mass spectrometry analysis revealed three sites of tyrosine phosphorylation, at Y74 on H1.5, at Y70 on H1.2 and at Y71 on H1.3 respectively, relative MS/MS spectra are reported in Figure 5. Interestingly, these sites are located in the highly conserved central globular domain of histone H1 [4].

To quantify the expression level of this peculiar PTM, we coupled 2D TAU Western blot with the anti-whole phospho-tyrosine antibody staining, considering that 2D TAU Western blot is able to resolve each H1 variants in a single spot and that H1 isoforms have a single tyrosine residue in their sequence as found by serendipity analysis,.

**Figure 6:**
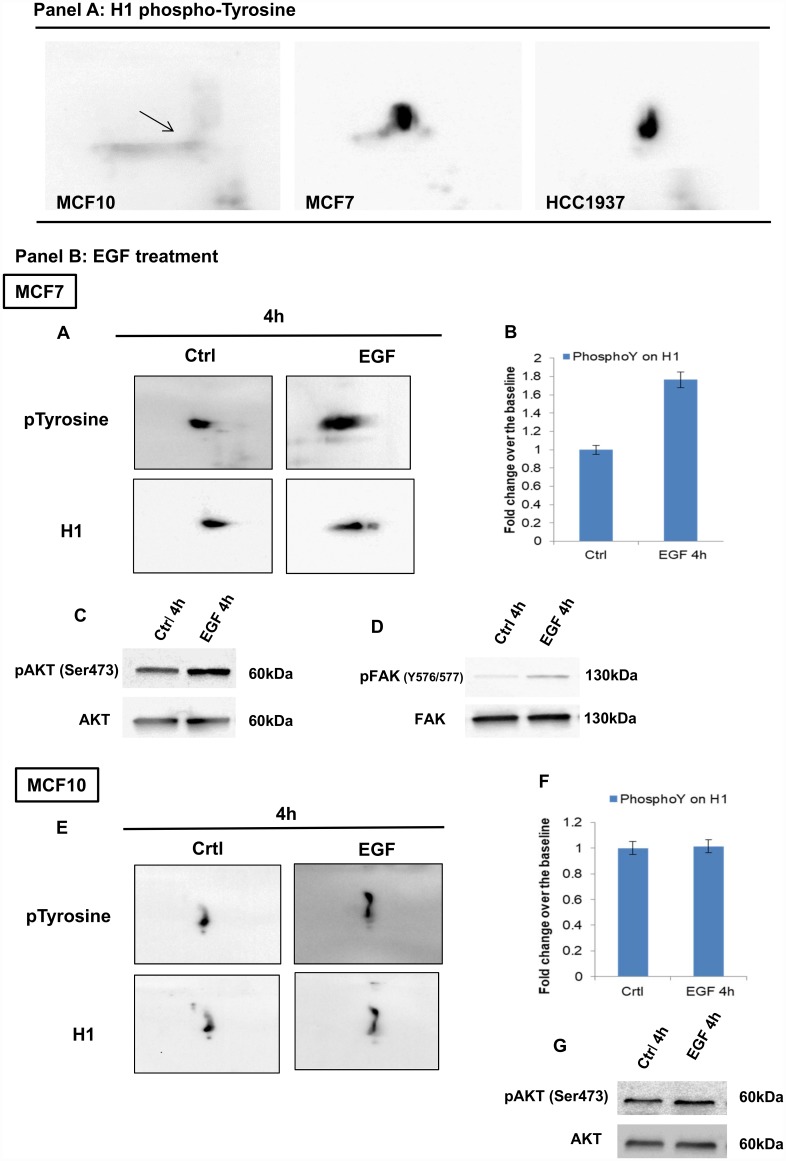
**Analysis of H1 histone tyrosine phosphorylation in breast and normal cancer cells, using 2D-TAU western blot.** (Panel **A**) 2D western blots showing tyrosine phosphorylation on the Histone H1 in MCF10, MCF7 and HCC1937 cells. Arrow indicates the region corresponding to the phosphorylated protein. Blot images were acquired using Alliance 2.7 (UVITEC, Eppendorf, Milan, Italy). Membranes signals were acquired concomitantly at 4 seconds. (Panel **B**) (**A**) 2D TAU western blot analysis of histone H1 tyrosine phosphorylation on MCF7 cells (acquisition time: 4’’) and (**E**) MCF10 cells (acquisition time: 20’’) following EGF stimulation. Lower panels indicate the relative normalization with H1 antibody; the relative densitometry analyses are shown in B for MCF7 cells and in F for MCF10 cell lines. The assays were repeated in three independent biological replicates Data are expressed as mean ± SEM (N =3). (**C**, **G**): Western blot analysis of pAKT and AKT levels on whole protein extracts from EGF treated MCF7 and MCF10 cells, respectively. (**D**) Western blot analysis of pFAK (Y576/577) levels on whole protein extracts from EGF treated MCF7.

Levels of tyrosine phosphorylation in H1 variants were significantly higher in breast cancer cells compared to normal cells suggesting a role of these modifications in breast cancer (Figure 6, panel A).

### LY294002, Troglitazone and PND1186 treatments reduce levels of H1 tyrosine phosphorylation in sporadic breast cancer cell lines

Recently, a paper, focused on global survey of phosphotyrosine signaling in lung cancer linked tyrosine histone phosphorylation with cellular proliferation [20].

In order to shed more light into biology of H1 tyrosine phosphorylation and to define correlations with cancer phenotype and progression, we carried out a set of experiments in which mitogenic pathways had been pharmacologically modulated.

Cell proliferation induced by recombinant epidermal growth factor (rEGF) produced in both normal and breast cancer cells, an increased tyrosine phosphorylation of H1, more evident in tumoral cells (MCF7) compared to normal cells (MCF10) (Figure 6, panel B and C).

**Figure 7:**
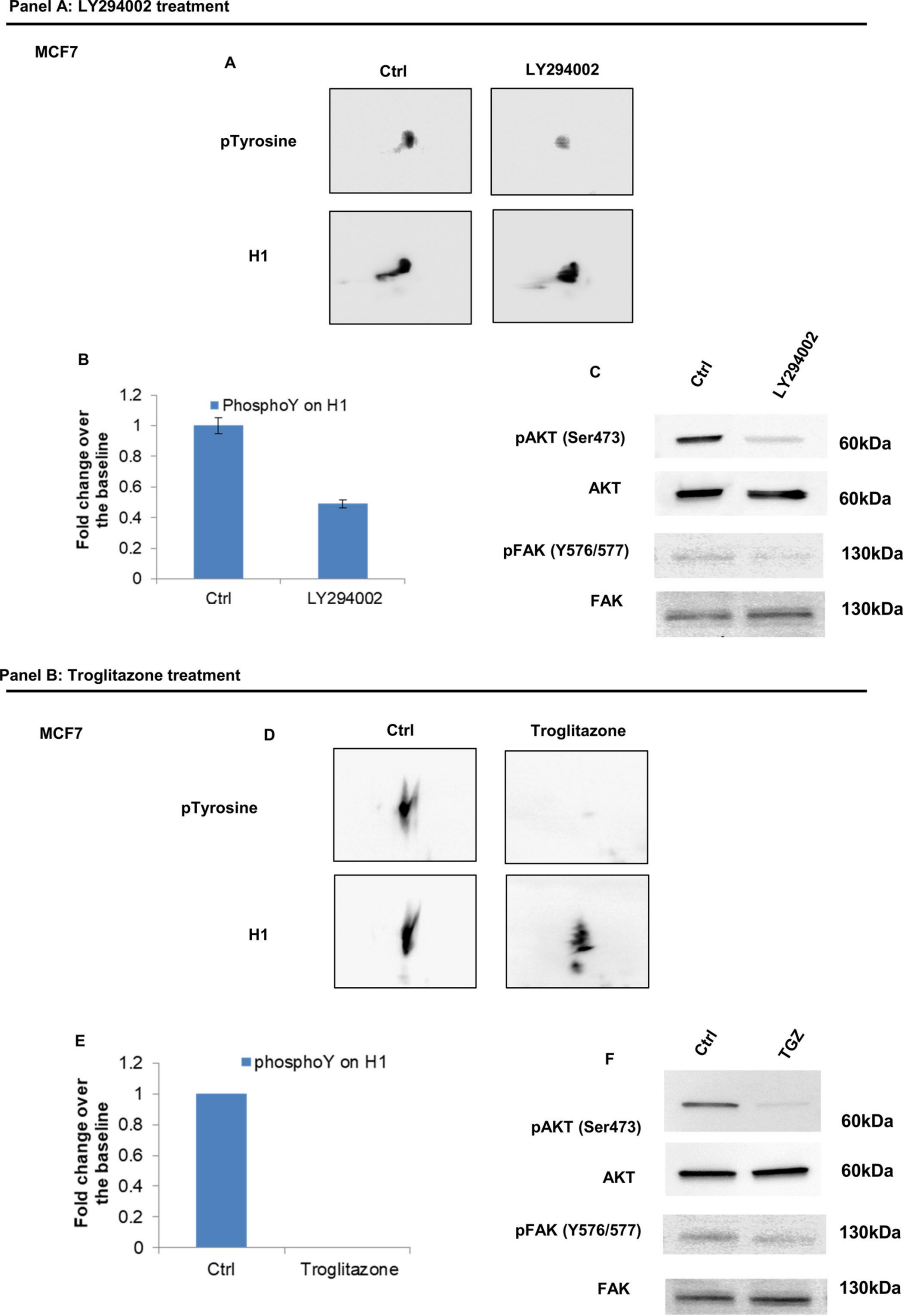
**Analysis of H1 histone tyrosine phosphorylation following LY294002 and Troglitazone treatments in MCF7 cells.** (Panel **A**) (**A**) 2D TAU Western blot analysis of histone H1 tyrosine phosphorylation level (upper panel) and relative normalization with H1 antibody (lower panel) after treatment of MCF7 with LY294002; All WB images were acquired in 4 seconds. (**B**) Densitometry analysis of H1 tyrosine phosphorylation spots; (**C**) Western blot analysis of pAKT, and pFAK (Y576/577) levels in protein extracts from LY294002 treated cells. Phospho-Akt (ser473) and pFAK (Y576/577) signals were normalized to the corresponding total Akt and total FAK, respectively. (Panel **B**) (**D**) 2D TAU Western blot analysis of histone H1 tyrosine phosphorylation level (upper panel) and relative normalization with H1 antibody (lower panel) after treatment of MCF7 with Troglitazone; All WB 2D images were acquired in 4 seconds. (**E**) Densitometry analysis of H1 tyrosine phosphorylation spots; (**F**) Western blot analysis of pAKT, and pFAK (Y576/577) levels in protein extract from Troglitazone treated cells. Phospho-Akt (ser473) and pFAK (Y576/577) signals were normalized agianst the corresponding total Akt and total FAK respectively. The assays were repeated in three independent biological replicates. Data are expressed as mean ± SEM (N =3).

Consistently, treatment by LY294002, a specific PI3K inhibitor [21, 22] and troglitazone (TGZ), a PPARγ agonist, known to exhibit anti-proliferative activity in breast cancer cells [23–25] both achieved a significant reduction of H1 tyrosine phosphorylation in MCF7 (Figure 7, panel A and B). Impaired tyrosine phosphorylation levels on H1 were assessed by 2D Western blot mapping.

Once tyrosine phosphorylation was correlated with proliferation, the *in silico* tool Phosphonet (http://www.phosphonet.ca) was used to analyze the consensus of histone that encloses phosphorylated tyrosine. Phosphonet database allows us to assess, that phosphosite Y74 of histone H1.5; Y70 of H1.2 and at Y71 of H1.3 were putative consensus of the Focal Adhesion Kinase (FAK), a tyrosine kinase capable of nuclear localization [26, 28]. To define its role in our system, we modulated FAK activity by a specific inhibitor, PND1186. PND1186, also known as VS-4718, is a reversible FAK inhibitor with an IC50 value of 100 nM in breast carcinoma cells [29, 30].

**Figure 8:**
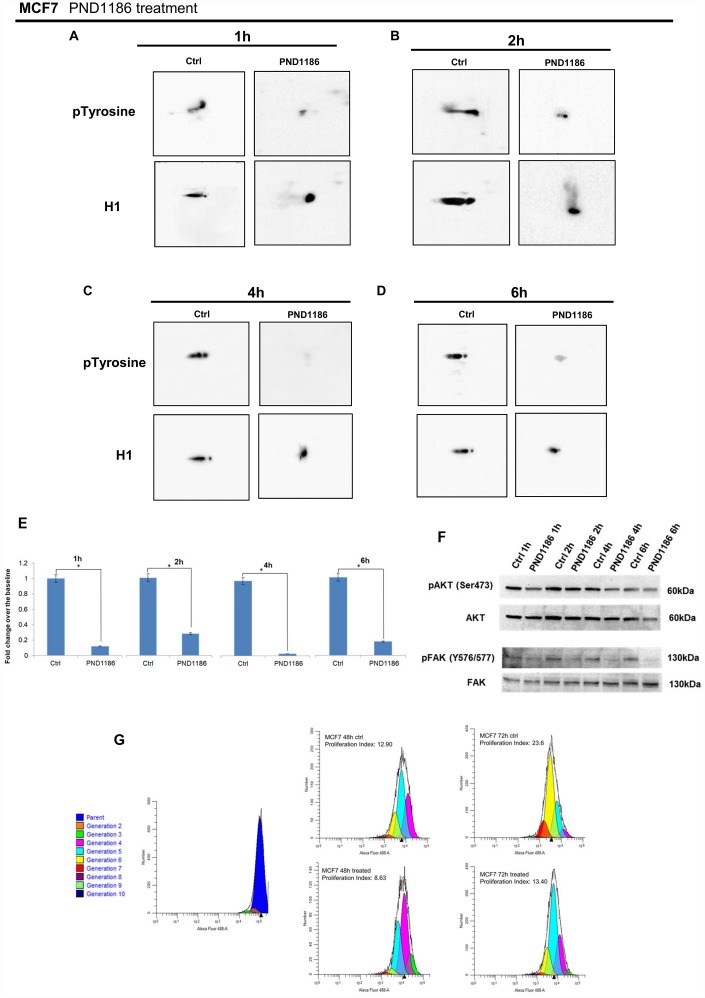
**Analysis of H1 histone tyrosine phosphorylation following PND1186 treatments in MCF7 cells.** 2D TAU Western blot analysis of histone H1 tyrosine phosphorylation (upper panel) and relative normalization with H1 antibody (lower panel) after PND1186 treatment; a time course of FAK inhibition was done at 1 hour (Panel **A**), 2 hours (Panel **B**), 4 hours (Panel **C**) and 6 hours (Panel **D**). All WB 2D images were acquired in 4 seconds. (Panel **E**) Densitometry analysis of H1 tyrosine phosphorylation spots; (panel **F**) Western blot analysis of pAKT, and pFAK levels in protein extracts from PND1186-treated cells. Phospho-Akt (ser473) and pFAK (Y576/577) signals were normalized against the corresponding total Akt and total FAK respectively. The assays were repeated in three independent biological replicates. Data are expressed as mean ± SEM (N =3), (**G**) Cells untreated and in presence of PND1186 300nM were cultured for 48 and 72 hours. Proliferation potential was detected, at single cell level, by CellTrace™ CFSE labeling. FACS analysis was performed at T_0_ (immediately after cell staining to define the parent population) and at 48 and 72 hours. Data were analysed by ModFit LT™ 4.0 software and the proliferation index has been generated for each sample.

Interestingly at 1 hour FAK inhibition induced a remarkable reduction of H1 tyrosine phosphorylation (Figure 8A). We performed a time course analysis and, as shown in Figure 8F, downregulation of H1 tyrosine phosphorylation had a timing suggestive of an involvement of FAK in controlling and/or catalyzing the reaction.

A cell proliferation assay allowed us to formalize the activity of PND1186 on (MCF7) proliferation of breast cancer cells. As shown in Figure 8G FAK inhibition decreases cell proliferation in a time dependent manner.

### LY294002, Troglitazone and PND1186 treatments reduce levels of H1 tyrosine phosphorylation in hereditary breast cancer cell lines

**Figure 9:**
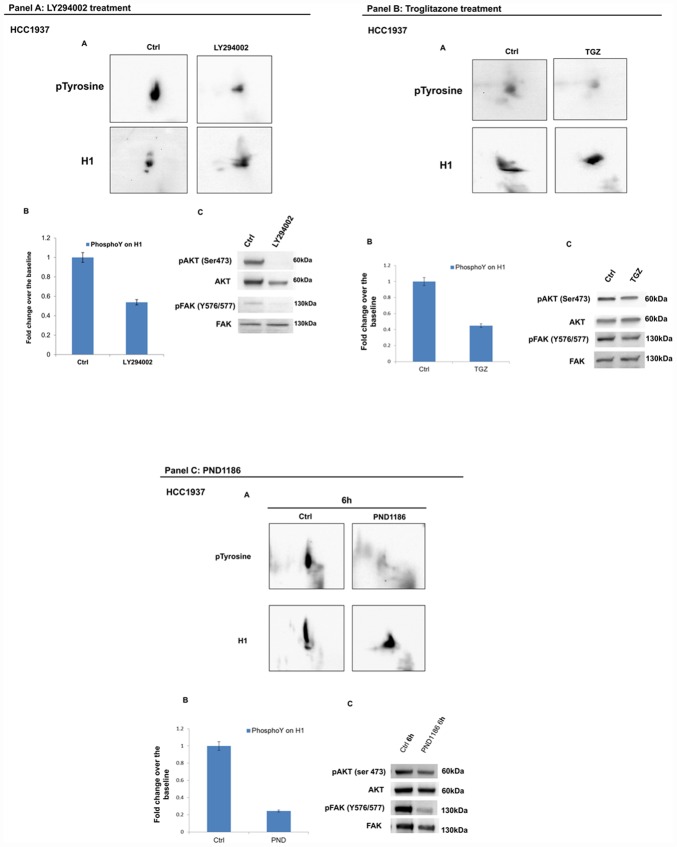
**Analysis of H1 histone tyrosine phosphorylation following LY294002, Troglitazone and PND1186 treatments in HCC1937 cells.** (Panel **A**) Analysis of H1 histone tyrosine phosphorylation following LY294002 treatments in HCC1937 cells. (**A**) 2D TAU Western blot analysis of histone H1 tyrosine phosphorylation level (upper panel) and relative normalization with H1 antibody (lower panel) after treatment of HCC1937 with LY294002; All WB 2D images were acquired in 4 seconds. (**B**) Densitometry analysis of H1 tyrosine phosphorylation spots; (**C**) Western blot analysis of pAKT, and pFAK (Y576/577) levels in protein extracts from LY294002 treated cells. Phospho-Akt (ser473) and pFAK (Y576/577) signals were normalized to the corresponding total Akt and total FAK respectively. (Panel **B**) Analysis of H1 histone tyrosine phosphorylation following Troglitazone treatments in HCC1937 cells. (**A**) 2D TAU Western blot analysis of histone H1 tyrosine phosphorylation level (upper panel) and relative normalization with H1 antibody (lower panel) after treatment of HCC1937 with Troglitazone; All WB 2D images were acquired in 4 seconds. (**B**) Densitometry analysis of H1 tyrosine phosphorylation spots; (**C**) Western blot analysis of pAKT, and pFAK (Y576/577) levels in protein extract from Troglitazone treated cells. Phospho-Akt (S473) and p-FAK (Y576/577) signals were normalized to the corresponding total Akt and total FAK respectively. (Panel **C**) Analysis of H1 histone tyrosine phosphorylation following PND1186 treatments in HCC1937 cells. (**A**) 2D TAU Western blot map of histone H1 tyrosine phosphorylation (upper panel) and relative normalization with H1 antibody (lower panel) after PND1186 treatment; All WB 2D images were acquired in 4 seconds. (**B**) Densitometry analysis of H1 tyrosine phosphorylation spots; (**C**) Western blot analysis of pAKT, and p-FAK levels in protein extract from PND1186 treated cells. Phospho-Akt (S473) and pFAK (Y576/577) signals were normalized to the corresponding total Akt and total FAK respectively. The assays were repeated in three independent biological replicates. Data are expressed as mean ± SEM (N =3).

The effects of LY294002, Troglitazone and PND1186 drugs were also assessed in HCC1937 breast cancer cells, a model of hereditary breast cancer. As shown in Figure 9, panel A, B and C, the three molecules are able to induce a significant reduction of H1 tyrosine phosphorylation levels not differently from what we observed in the sporadic model.

### Immunoprecipitation analysis reveals an interaction between nuclear FAK and histone H1

**Figure 10:**
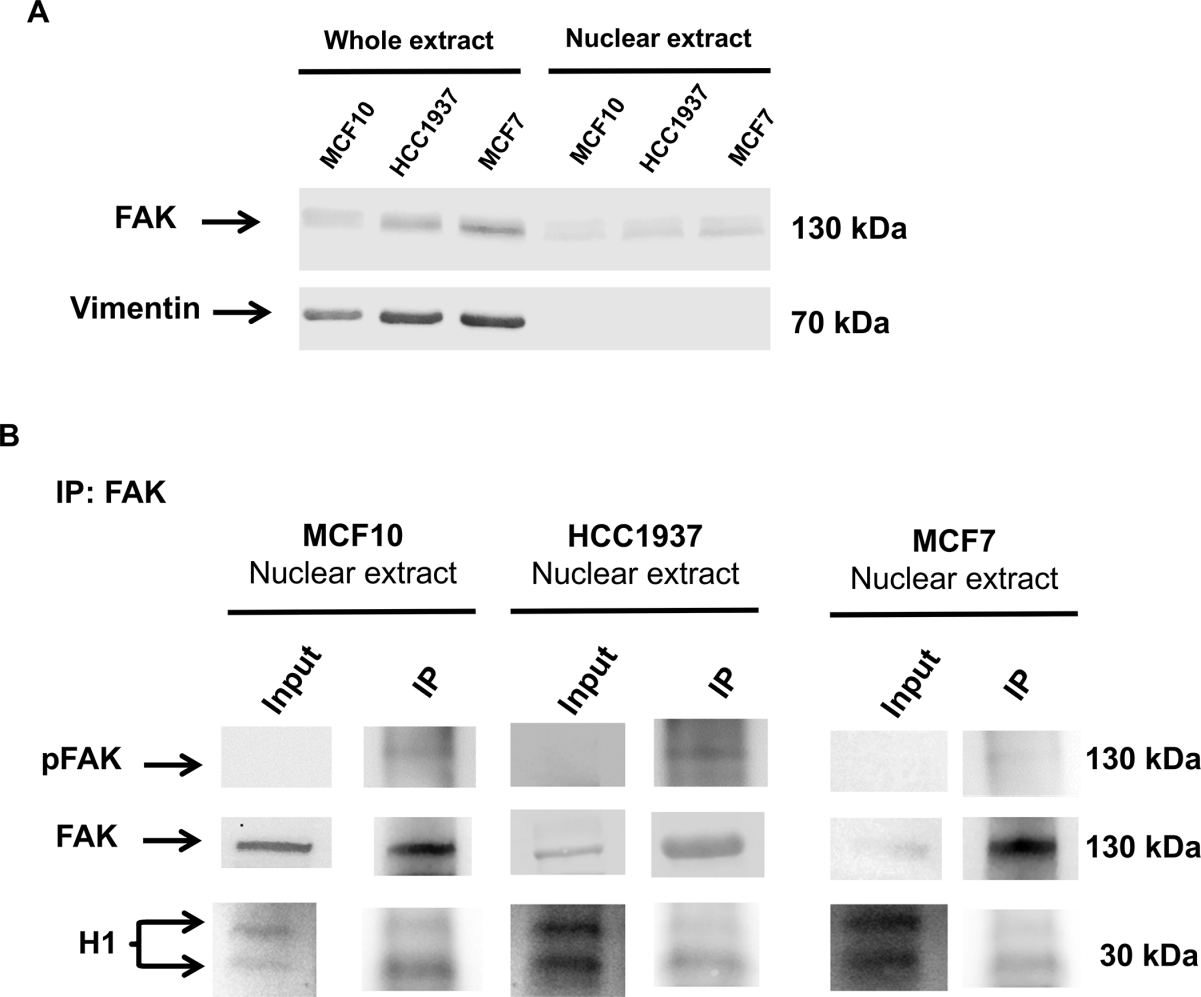
**Immunoprecipitation analysis of nuclear FAK and histone H1.** Immunoprecipitation (IP) analysis demonstrated that nuclear FAK associates with H1 histone. IP was carried out with anti-FAK antibody (3285, Cell signaling), followed by immunoblotting with anti-FAK (D2R2E, Cell signaling), anti-pFAK (Y576/577) and anti-H1 antibodies. IP experiments were done on MCF7, HCC1937 and MCF10 cell lines. The images are representative of three independent biological replicates. Panel **A** shows the levels of FAK in whole and nuclear extracts; Vimentin blot assess that nuclear extracts are not contaminated by cytoplasmic fraction.

The ability of FAK to directly interact with the histone H1, was investigated by co-immunoprecipitation experiments. We incubated the nuclear protein extract from MCF10, MCF7 and HCC1937 cells with an anti-FAK antibody. The immunoprecipitated fraction was assayed with antibodies against histone H1 and pFAK. Interestingly pFAK and Histone H1 co-immunoprecipitated, demonstrating that pFAK and H1 are potentially capable of a direct interaction (Figure 10). The levels of FAK in whole and nuclear extracts are shown in Figure 10A; Vimentin blot is used to assess that nuclear extracts are not contaminated by cytoplasmic fraction.

### Immunofluorescence analysis assess the co-localization of FAK and Histone H1 in the nuclei of proliferating breast cancer cells

**Figure 11:**
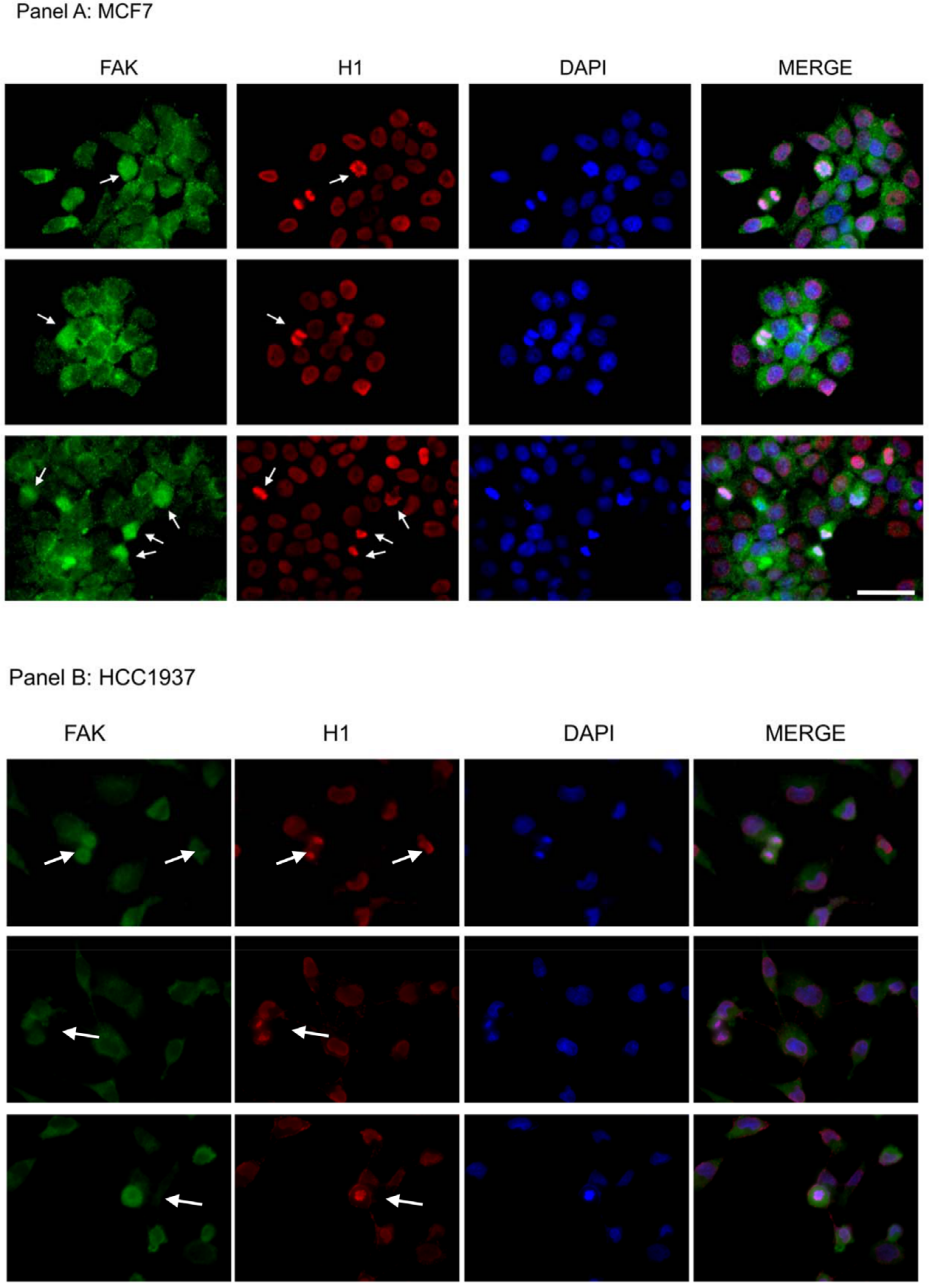
**Assessment of FAK and H1 localization in MCF7 and HCC1937 cell lines.** Immunofluorescence analysis of an asynchronous MCF7 cell population stained with antibodies against FAK (green) and H1 (red). Images of the cells captured at different stages of mitosis revealed FAK co-localization with H1 (white arrows). The nuclei were counterstained with DAPI (blue) and merge is also shown. Scale bar: 50 μm. The images are representative of three independent biological replicates.

To further support the hypothesis of a direct interaction, we investigated the localization of endogenous FAK and histone H1 in the nuclei of MCF7 and HCC1937 cells by immunofluorescence analysis. As expected FAK protein had a predominant cytoplasmic localization, with a weak nuclear signal during the interphase, however FAK strongly marked the mitotic spindle in dividing cells, co-localizing with H1 during metaphase, anaphase and telophase (Figure 11A and 11B). These results, although not conclusive, enforce the hypothesis of a FAK direct involvement in histone H1 phosphorylation.

## DISCUSSION AND CONCLUSIONS

Epigenetics plays a key role in physiological processes as well as in the onset and progression of several pathologies. Recently, attention focused on epigenetic phenomena and their association with cancer. Moreover, unlike genetic alterations, epigenetic changes are a reversible phenomenon and therefore potentially druggable [3].

In addition to DNA methylation, histone PTMs are main actors in epigenetic regulation of gene expression. By acting individually or in combination, histone marks modulate the transcriptional state of chromatin and by assembling and disassembling nucleosomes allow or not the binding of transcription factors and ultimately, the building up of the transcriptional machinery.

There are several histone PTMs, with specific biological and pathophysiological implications, whose altered expression has been associated with cancer. Conversely, many other PTMs have roles still unknown.

In our work, we investigated the pattern of histone PTMs in normal and breast cancer cell lines by 2D TAU electrophoresis coupled with mass spectrometry.

The comprehensive analysis of the unmapped PTMs was performed by two-dimensional Western blotting using anti-Y-phospho antibody.

The pattern of PTMs of canonical histones and their variants allowed us to evaluate and to identify differentially expressed isoforms among the three breast cell types used. Particularly, by overlapping immunoblotting results with mass spectrometry data, we found peculiar tyrosine phosphorylations at residues Y74 of histone H1.5; at Y70 in H1.2 and at Y71 in H1.3.

Our data clearly demonstrate that levels of H1 tyrosine phosphorylation are much higher in both the analyzed breast cancer cells (MCF7 and HCC1937) compared to the immortalized normal epithelial cell line MCF10. By modulating mitogenic pathways, we established a correlation between H1 tyrosine phosphorylation and cell proliferative status, enforcing the notion of a role of phosphorylated histones in the definition of the tumor phenotype. We give evidence that EGF treatment was able to induce a significant increase of tyrosine phosphorylation in H1, much more evident in MCF7 cells than in MCF10 cells. Next, we analyzed the effects that modulation of three signaling pathways, known to be altered in cancerogenesis, PI3K, PPARγ and FAK, could have on phosphorylation in H1.

Phosphoinositide 3-kinase (PI3K) and its downstream mediator AKT are activated in many types of cancer and regulate many processes including proliferation, migration, apoptosis, differentiation and cell adhesion [21, 22]. Our data disclosed that the treatment with LY294002, a PI3K inhibitor, leads to a 50% reduction of tyrosine phosphorylation in H1 in both tumor cell types, suggesting that the kinase regulating this phosphorylation must be a downstream effector of PI3K signaling.

Furthermore, we treated tumor cells with Troglitazone, a PPARγ agonist whose anti-tumor properties in breast cancer have been extensively reported [23]. Troglitazone belongs to Thiazolidinediones (TZDs) antidiabetic drugs and has antiproliferative effects [24] by both receptor dependent and independent actions. Peroxisome proliferator-activated receptor γ (PPARγ) is a member of nuclear ligand-dependent transcription factor whose activation leads to growth inhibition in human breast cancer cells. Activation of PPARγ controls cell migration by upregulating the expression of PTEN, that leads, in turn, to a decrease of FAK and paxillin phosphorylation [25]. In our study, treatment with Troglitazone induces a significant reduction of tyrosine phosphorylation levels on histone H1.

These results, together with the search of phosphorylation consensus, allowed us to focus the attention on Focal adhesion kinase (FAK), as a candidate for the H1 tyrosine phosphorylation. FAK is an evolutionarily conserved non-receptor tyrosine kinase that plays important roles in specific cellular functions such as adhesion, migration, invasion, polarity, proliferation and survival [26, 27]. The auto-phosphorylation of FAK at Y397 is the first step for its activation and creates a binding site for Src. Thus, Src phosphorylates several sites of FAK, including the tyrosine 576 and 577 within the central kinase domain and positively regulates FAK kinase activity. AKT associates with FAK and directly modulates its expression through phosphorylation at several serine and threonine residues [31]. FAK is overexpressed in several cancers; specifically, high levels of FAK were found in breast cancer tissues and correlate with cancer progression and lymph node positivity [26]. Under normal conditions, FAK is typically a cytoplasmic kinase, but it can shuttle into the nucleus under appropriate stimuli in several cell types [28–32]. In the nuclei, FAK promotes p53 ubiquitination, acting as a scaffold [33]. Moreover, FAK has a role in histone PTMs promoting H3K27me3 through the regulation of zeste homolog 2 enhancer (EZH2) [34]. Nuclear FAK is associated with chromatin in squamous cell carcinoma (SCC) and interacts with a number of transcription factors and regulators [35, 36], interfering with GATA4 transcription factor and controlling chromatin structure [28].

Treatment with PND1186, a selective FAK inhibitor, induces in our cell systems a significant reduction of H1 tyrosine phosphorylation, detectable very early, as expected if FAK directly acts on the substrate.

The ability of FAK to directly interact with the histone H1 was supported by their co-immunoprecipitation, and co-localization during the interphase of cell cycle assessed immunofluorescence analysis.

In conclusion, this work offers a robust method for a comprehensive cover of PTMs occurring in histones in normal, sporadic and hereditary breast cancer cell lines. It proposes H1 tyrosine phosphorylation as novel PTM with a potential, relevant role in breast tumorigenesis and it candidates FAK as the kinase probably involved in H1 phosphorylation suggesting a novel key role of FAK in controlling post-translational events relevant in breast cancer proliferation.

## MATERIALS AND METHODS

### Cell cultures

Human breast cancer cell line MCF7 (ATCC, Manassas, USA) was grown in Dulbecco’s modified Eagle’s medium (DMEM) (Sigma Aldrich, Saint Louis, Missouri, USA) supplemented with 10% (w/v) fetal bovine serum (FBS) (Sigma Aldrich), 100 mg/ml streptomycin and 100U/ml penicillin (Sigma Aldrich). MCF10 mammary epithelial cell line (ATCC, Manassas, USA) was grown in MEGM Mammary Epithelial Cell Growth Medium (Lonza, Walkersville, MD) supplemented with 20 ng/ml epidermal growth factor (Lonza), 0,5 μg/ml hydrocortisone (Lonza), 100 ng/ml cholera toxin (Sigma Aldrich) and 10 μg/ml insulin (Lonza). HCC1937 cell line was homozygous for the BRCA1 5382C mutation and was used as a model of hereditary breast cancer. They carried a mutation on TP53 gene with wild-type allele loss and a homozygous deletion of PTEN gene. HCC1937 cells (ATCC) were grew in RPMI medium (ATCC) supplemented with 20% (w/v) fetal bovine serum (FBS) (Sigma Aldrich), 100 mg/ml streptomycin and 100U/ml penicillin (Sigma Aldrich). The rate of cell in each cell cycle was assessed by FACS analysis. Histones used for the matching, were extracted from cell lines having a comparable distribution among the cell cycle phases.

Cell cycle was analyzed by propidium iodide (PI) staining and flow cytometry. Briefly, 1x106 cell, were fixed by resuspension in 500 μl of cold 70% ethanol under continuous gentle vortexing and left at 4°C 30 minutes or kept overnight at −20°C. Cells were then recovered by centrifugation, washed twice in PBS and incubated for 1h at room temperature in 1 ml PBS containing propidium iodide (20 μg/ml), NP40 0.1% and ribonuclease (40 μ/ml). Samples were analyzed by a BD™ LSRFortessa™ X-20 Flow Cytometer with 488-nm excitation and a 610/20nm bandpass emission filter. FlowJo™ software was used for data analysis.

### Protein extraction

### Histones extraction from mammalian cell culture

Acid-extraction of histone proteins was performed according to the protocol of Shechter et al. [9]. Cells were centrifuged at 1000xg for 5 min and resuspended in hypotonic lysis buffer (10 mM Tris-Cl pH 8.0, 1 mM KCl, 1.5 mM MgCl_2_, 1 mM DTT, 1 mM phenyl-methylsulfonyl fluoride, PMSF) supplemented with protease and phosphatase inhibitor cocktail (Halt Protease Inhibitor Cocktail/ Halt Phosphatase Inhibitor Cocktail, Thermo Fisher Scientific Inc.) at a density of 5 × 10^6^ cells ml^−1^ Cells lysate was incubated for 30 min on rotator at 4°C. Nuclei were isolated by centrifugation at 10000 ×g for 10 min at 4°C. Histones were extracted by incubation with 0.4 N H_2_SO_4_ on ice. Proteins were precipitated using trichloroacetic acid (TCA). Histone pellets were washed twice with ice-cold acetone, lyophilized and then solubilized in sterile H_2_O. The extraction was verified by 1D-TAU GEL. Resulting gel was stained with EZBlue Gel Staining Reagent (Sigma Aldrich).

### Whole protein extraction

Cells lines were washed with PBS and lysed at 0°C for 30 min using lysis buffer (15 mM Tris pH7.5, 120mM NaCl, 25mM KCl, 0.5% Triton X-100, supplemented with protease and phosphatase inhibitor cocktail). Cell lysate was sonicated at 4 °C for 10 sec and subsequently centrifuged at 15000× g for 20 min. Supernatant was carefully removed and protein content was measured by the Bradford method (BioRad, Hercules, CA) [37]; and the supernatants were stored at 80°C.

### Isolation of nuclear and cytosolic fractions

Cells were collected with 1ml of hypotonic lysis buffer (10 mM Tris-Cl pH 8.0, 1 mM KCl, 1.5 mM MgCl2, 1 mM DTT, supplemented with protease and phosphatase inhibitor cocktail) and incubated for 30 min on rotator at 4°C. Cell lysate was centrifugated at 10000×g for 10 min at 4°C to isolate nuclei [38, 39]. Cytosol was supplemented with 1% Triton X-100. Both fractions were incubated on ice for 30 minutes and then centrifuged at 15000xg for 20 min at 4°C. Protein concentration was determined using the Bradford Protein Assay (Bio-Rad) according to the manufacturer’s instructions with BSA as standards.

### 2D Gel electrophoresis

### First dimension: triton-acid-urea (TAU) gel electrophoresis

TAU gel was prepared according to the protocol of Shechter et al. [11]. Thirty-five μg of lyophilized histone powder was dissolved in acidic sample buffer (6 M Urea, 5% glacial acetic acid, 0.02% Pyronin Y) and loaded on the gel. The gel was run in 5% acetic acid solution at the voltage of 25 V for about 17h. The gel was then stained with EZBlue Gel Staining Reagent (Sigma Aldrich) to evaluate proteins separation.

### Second dimension: SDS-PAGE gel electrophoresis

Lanes of TAU gel, containing resolved histones, were cut and transferred to the top of a 12% SDS polyacrylamide gels (Mini-PROTEAN® TGX™ Precast Gels, IPG Well) for the separation based on molecular weight. Second dimension was run at 80 V until the bromophenol blue dye front reached the end of the gels [12]. Gels were stained with EZBlue Gel Staining Reagent (Sigma Aldrich) or MS compatible silver staining procedure. The analysis was performed in triplicate. Gel image analysis was carried out using the Image Master 2D-Platinum software 6.0 (GE Healthcare) [40, 41].

### In-gel tryptic digestion

Protein spots, obtained from 2D TAU/SDS gels, were manually excised, destained, and dehydrated in acetonitrile. They were then rehydrated and digested in trypsin solution by overnight incubation at 37°C. After drying the organic solvent, the tryptic peptides were purified by Pierce C18 Spin Columns (Thermo Fisher Scientific Inc.) for desalting before mass spectrometric analysis. The purified peptides were eluted with 40μL of 70% acetonitrile and dehydrated in a vacuum evaporator [42, 43].

### Nanoscale LC-MS/MS analysis

LC-MS/MS analysis was performed using an Easy LC 1000 nanoscale liquid chromatography (nanoLC) system (Thermo Fisher Scientific, Odense, Denmark). The analytical nanoLC column was a pulled fused silica capillary, 75 μm i.d., in-house packed to a length of 10 cm with 3 μm C18 silica particles from Dr. Maisch (Entringen, Germany). The peptide mixtures were loaded at 500 nL/min directly onto the analytical column. A binary gradient was used for peptide elution. Mobile phase A was 0.1% formic acid, 2% acetonitrile, whereas mobile phase B was 0.1% formic acid, 80% acetonitrile. Gradient elution was achieved at 350 nL/min flow rate, and ramped from 0% B to 30% B in 15 minutes, and from 30% B to 100% B in additional 5 minutes; after 5 minutes at 100% B, the column was re-equilibrated at 0% B for 10 minutes before the following injection. MS detection was performed on a quadrupole-orbitrap mass spectrometer Q-Exactive (Thermo Fisher Scientific, Bremen, Germany) operating in positive ion mode, with nanoelectrospray (nESI) potential at 1800 V applied on the column front-end via a tee piece. Data-dependent acquisition was performed by using a top-4 method with resolution (FWHM), AGC target and maximum injection time (ms) for full MS and MS/MS of, respectively, 70,000/17,500, 1e6/5e5, 50/400. Mass window for precursor ion isolation was 2.0 m/z, whereas normalized collision energy was 30. Ion threshold for triggering MS/MS events was 2e4. Dynamic exclusion was 15 s [43, 44].

### MS data processing and database searching

The acquired raw data files were preprocessed with Proteome Discoverer 1.4 (Thermo Fisher Scientific, Bremen, Germany). MS/MS data were searched on the Human UniProt database. False discovery rate (FDR) of peptide identifications was estimated using the “Target-decoy PSM validator” node in Proteome Discoverer [45]. The following search parameters were used: MS error tolerance: 5 ppm; MS/MS error tolerance: 0.02 Da; enzyme specificity: trypsin; maximum number of missed cleavages: 2; taxonomy Human; fixed modifications: Carbamidomethylation (C);; variable modification: Oxidation (M), Acetyl (K), Methyl (K), Dimethyl (K), Trimethyl (K), Methyl (R), Dimethyl (R), Deamidated (R) and Phosphorylation (STY). Protein hits based on two successful peptide identifications (Xcorr> 2.0 for doubly charged peptides, >2.5 for triply charged peptides, and >3.0 for peptides having a charge state >3) were considered valid. All the MS/MS spectra, referred to modified peptides, were carefully verified by manually validation.

### Western blot analyses

For 1D Western blot analysis, five μg of each histone sample was resolved by 15% SDS-PAGE and electrotransferred to a nitrocellulose membrane with a Trans-blot turbo system (Biorad). Membranes were incubated using the following primary antibodies: H4K16ac (1:1000; Cell signaling E2B8W), H4K20me3 (1:1000; Cell signaling D84D2), H3K9ac (1:1000; Cell signaling C5B11), H3K9me2-3 (1:1000; Cell signaling 6F12) and H3K18ac (1:1000; Cell signaling D8Z5H). Only for SirT1 analysis, fifty μg of total cells proteins extract were loaded in a 4–20% SDS-PAGE (Biorad), transferred to a membrane and blotted with the SirT1 antibody (1:1000; Cell signaling 1F3). To *ensure equal* loading of proteins was used a goat polyclonal anti-*γ*-*Tubulin* antibody (C-20) (1:1000; sc-7396, Santa Cruz Biotechnology).

For 2D Western blot analysis, equal amounts of histone extracts were resolved by 2D TAU/SDS gel. To minimize gel to gel variation and ensure a reliable comparison among analyzed samples second dimension for each sample was run on Mini-PROTEAN(R) TGX™ Precast Gels, IPG Well (12%). Resulting gel were transferred to nitrocellulose membranes with a Trans-blot turbo system (Biorad) using Trans-Blot(R) Turbo™ Mini Nitrocellulose Transfer Packs. Membranes were probed using the Phospho-Tyrosine (1:2000; Cell signaling P-Tyr-100) antibody. Phospho- Tyrosine signals were normalized to the corresponding total H1 (1:1000; Abcam ab71594).

To ensure equal protein loading, membranes were incubated with red ponceau solution (P7170 Sigma-Aldrich). Images were acquired using Image scanner II (GE Helthcare). Membrane image were visualized using image master 2D platinum.

### Cell treatments

Cells at 50% confluence were serum starved for 24 h. Cell treatments were performed using recombinant human EGF (PeproTech) C_f_: 50ng/ml, LY294002 (Sigma Aldrich) C_f_: 30μM, Troglitazone (MedChemExpress) C_f_: 50μM and PND1186 (MedChemExpress) C_f_: 300nM.

Flow cytometry experiment was done to assess the rate of cell cycle phases after treatment, cells were collected, fixed with 70% ethanol, stained with propidium iodide solution and analyzed by flow cytometry.

Histone pattern was analyzed by 2D TAU Western blot using Phospho-Tyrosine (1:2000; Cell signaling P-Tyr-100) and H1 (1:100; Abcam ab71594) antibodies.

Whole proteins extract and nuclear protein fraction were analyzed by Western blotting using the following primary antibodies: pAKT (Ser473) (1:1000; D9E), AKT (1:1000; 9272), pFAK (Tyr397) (1:200; D20B1), pFAK (Tyr576/577) (1:500) and FAK (1:1000; all Cell signaling).

### Densitometric analysis

Secondary antibodies signal was revealed by Pierce ECL Western Blotting Substrate (Thermo scientific). Images were acquired using the UVIsoft Image quantification software. Signals intensities were assessed by densitometry using the Alliance 4.7 software. For each 2D western blot the exposure times were fixed to 10 seconds for phosphotyrosine signal detection and 5 seconds for total histone H1 signal detection.

Densitometric analysis of 2D Western Blotting spots was performed by Image Master 2D- Platinum software 6.0 (GE Healthcare). Data were analyzed using Excel spreadsheet (Microsoft office), and expressed as mean ± SEM (N =3), where SEM represents the standard error of the mean and N indicates the number of experimental repeats. Statistical analysis was performed using one-way ANOVA, followed by Dunnett's multiple comparisons test. Differences were considered significant when P≤0.05.

### Cell proliferation assay

CellTrace™ CFSE Cell Proliferation assay has been performed in accordance with manufacturer instruction. Cell labeling was carried out as follows. CellTrace™ DMSO stock solution was diluted in phosphate-buffered saline (PBS) at the working concentration of 5μM. Cell pellet was obtained by centrifugation and cells were gently resuspended in prewarmed (37°C) PBS containing the dye and incubated for 20 minutes at room temperature protected from light. Cells were then washed twice with five times the original staining volume by culture medium containing FCS to remove any free dye remaining in the solution. Cells were finally plated in fresh, pre-warmed complete culture medium, analyzed, treated and cultured as indicated. FACS analysis was performed using a BD™ LSRFortessa™ X20 Flow Cytometer with 488-nm excitation and a 530/30-nm bandpass emission filter. Data were analyzed and proliferation index, defined as the total number of divisions divided by the number of cells that went into division, was generated, by ModFit LT™ 4.0 software.

### Immunoprecipitation analysis

Four hundred μg of nuclear protein extracts from untreated cells was subjected to immunoprecipitation with FAK antibody (Cell signaling, 3285). After overnight incubation at 4°C, protein A/G PLUS-Agarose beads (Santa Cruz Biotechnology) were added to the mixture and incubated for 4h at 4°C. Beads were washed tree times and boiled for 5 minutes. Immunocomplexes were resolved on 4-20% SDS gel, blotted using a Trans-blot turbo system (Biorad) for 30 minutes at 30V, 1.0A and then subjected to immunoblot analysis using the following antibodies: pFAK (Tyr397) (1:200; D20B1), FAK (D2R2E, Cell signaling)(1:1000) and H1 (1:100; Abcam ab71594).

The assays were repeated in three independent biological replicates. Whole and nuclear extract (100 μg each) were resolved by pre cast SDS-PAGE (Any kD™ Mini-PROTEAN Precast Protein Gels, Biorad) and electrotransferred to a nitrocellulose membrane with a Trans-blot turbo system (Biorad). Membranes were incubated using the following primary antibodies: FAK (D2R2E, Cell signaling)(1:1000), Vimentin (5741, Cell signaling, 1:1000). The detection of a primary antibody was done with antirabbit horseradish peroxidase-conjugate secondary antibodies (Cell Signaling). Blots were developed using the SuperSignal West Femto ECL substrate (Pierce, Thermo Fisher Scientific Inc., Bremen, Germany). Images were acquired using Alliance 2.7 (UVITEC, Eppendorf, Milan, Italy)

### Immunofluorescence

Cells were fixed with 4% paraformaldehyde (Sigma-Aldrich) for 30 min. After permeabilization with 0.3% Triton X-100 (Sigma-Aldrich) in phosphate buffered saline (PBS) for 15 min, the cells were blocked with 10% FBS (Biowest) and 0.1% Triton X-100 in PBS for 1 h at room temperature and then incubated with H1 (1:100, Abcam ab71594) and FAK (1:100, Cell Signaling, 3285) primary antibodies diluted in PBS containing 3% FBS. Goat anti-mouse Alexa-Fluor-647 (A-21235, Life Technologies) and goat anti-rabbit Alexa-Fluor-488 (A-11008, Life Technologies) were used as the secondary antibodies at a concentration of 2 μg/ml in PBS containing 1% FBS for 45 min at room temperature. Nuclei were stained with DAPI (4',6-diamidino-2-phenylindole). Slides were mounted with fluorescent mounting medium (Dako Cytomation) and images were acquired with DMi8 Leica microscope. The assays were repeated in three independent biological replicates

## Supplementary Material

Supplementary Figures
