# Supplementary material for: Histone proteomics reveals novel post-translational modifications in breast cancer
Source: Aging (Albany NY). 2019 Dec 8;11(23):11722–55. doi: 10.18632/aging.102577 (PMC6932915; doi:10.18632/aging.102577)
Supplement: Supplementary Figures [file aging-11-102577-s001..pdf]

# SUPPLEMENTARY FIGURES

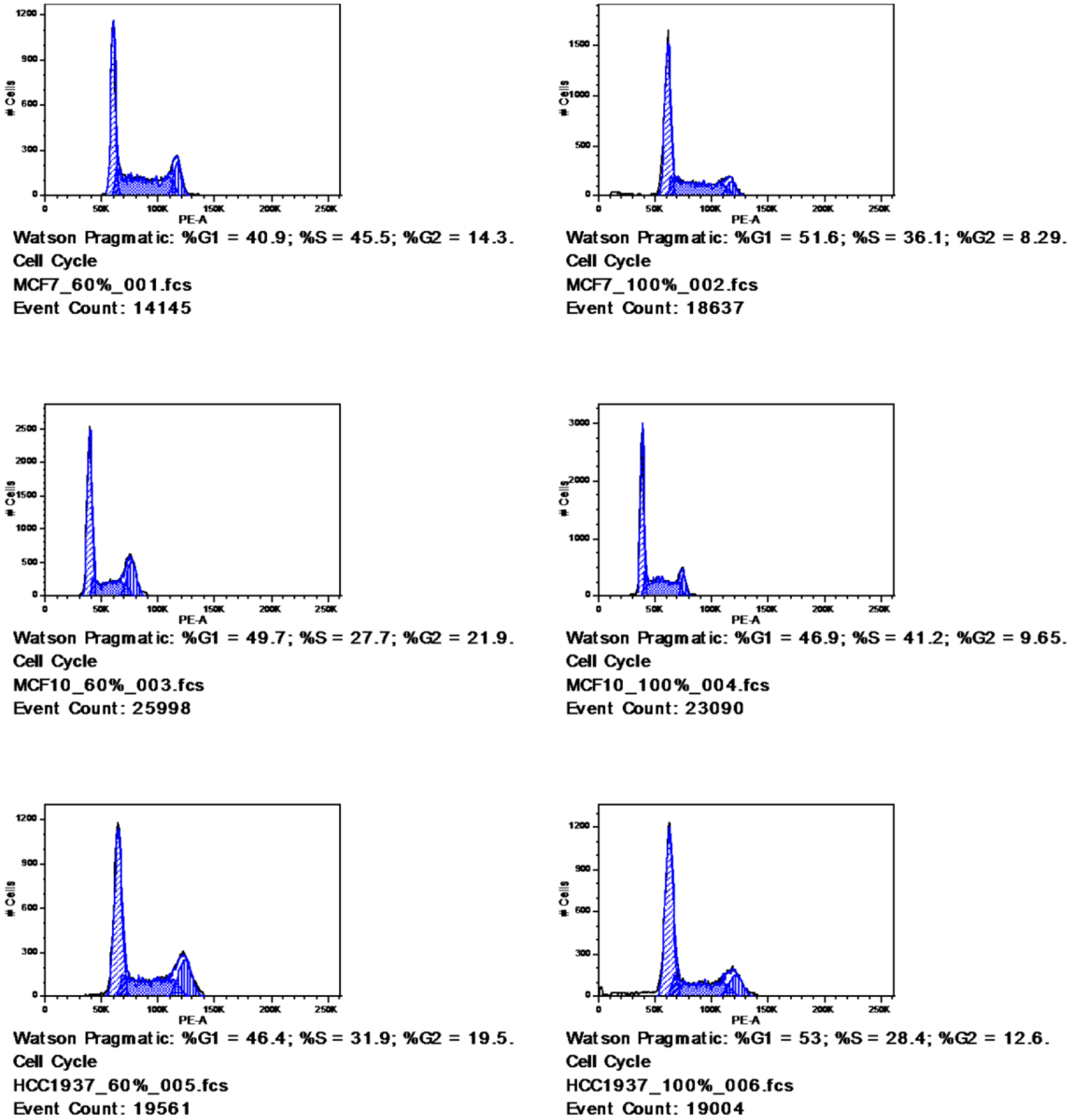


**Supplementary Figure 1. Cell cycle analysis.** $1\times10^6$ cell, were fixed by resuspention in 500 µl of cold 70% ethanol under continuous gentle vortexing and incubated at 4°C 30 minutes or kept overnight at −20°C. Cells were then recovered by centrifugation, washed twice in PBS and incubated for 1h at room temperature in 1 ml PBS containing propidium iodide (20 µg/ml), NP40 0.1% and ribonuclease (40 µ/ml). Samples were analysed by a BD™ LSRFortessa™ X-20 Flow Cytometer with 488-nm excitation and a 610/20nm bandpass emission filter. FlowJo™ software was used for data analysis.

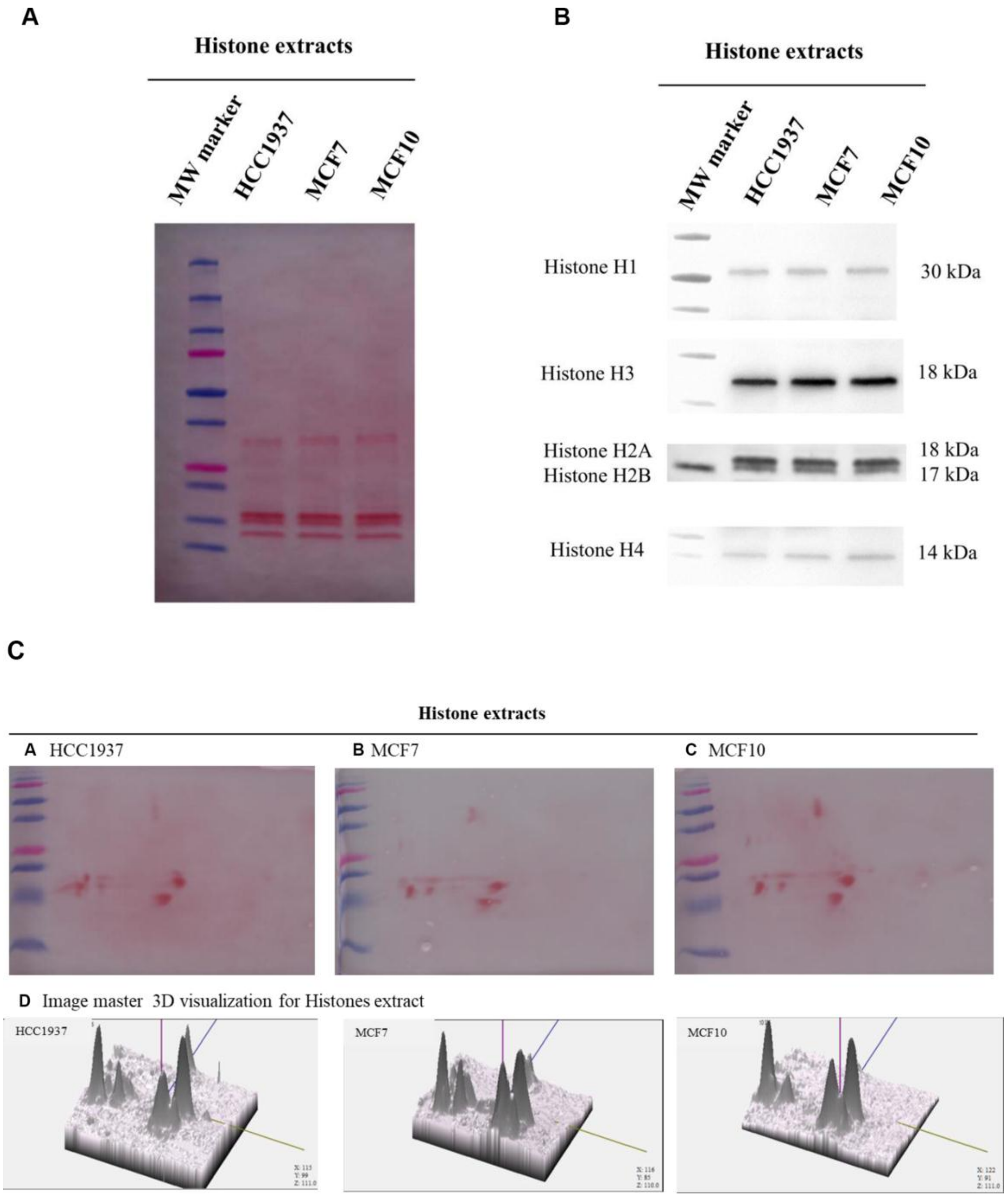


**Supplementary Figure 2. Western blot detection of Histone proteins.** (**A**) Red ponceau Staining of membrane. Histones extracts (5 micrograms) were resolved using 15% SDS-PAGE and electrotransferred to a nitrocellulose membrane with a Trans-blot turbo system (Biorad). Membranes were stianed using red ponceau solution (P7170 Sigma-Aldrich). Nitrocellulose membrane was destained using PBS 1X and used for immunologically detection. (**B**) Immunoblotting. Membranes were incubated using the following primary antibodies: Histone H1 (1:1000; Abcam ab71594), Histone H2B (D2H6) (1:1000; Cell signaling 112364), Histone H2A (1:1000; Cell signaling 2578), Histone H3 Antibody (1:1000; Cell signaling 9715), Histone H4 (1:1000; Cell signaling 2592). (**C**) 2D Western Blot. Red ponceau Staining of membranes and image master analysis. For 2D Western blot analysis, equal amounts of histone extracts were resolved by 2D TAU/SDS gel. To minimize gel to gel variation and ensure a reliable comparison among analyzed samples second dimension for each sample was run on Mini-PROTEAN® TGX™ Precast Gels, IPG Well (15%). Resulting gel were transferred to nitrocellulose membranes with a Trans-blot turbo system (Biorad) using Trans-Blot® Turbo™ Mini Nitrocellulose Transfer Packs. To ensure equal protein loading, membranes were incubated with red ponceau solution (P7170 Sigma-Aldrich). Images were acquired using Image scanner II (GE Helthcare). Membrane image were visualized using image master 2D platinum. 3D visualization tool testify the loading of equal amount for each Histone extract (panel D).

**$K_{Dimethyl}$ASGPPVSELITK** **H1.2K33 – H1.3K34**

Sequence: KASGPPVSELITK, K1-Dimethyl (28.03130 Da)
Charge: +2, Monoisotopic m/z: 677.90155 Da (+0.86 mmu/+1.26 ppm), MH+: 1354.79582 Da, RT: 19.59 min,
Identified with: Sequest HT (v1.3); XCorr:3.93, Ions matched by search engine: 0/0
Fragment match tolerance used for search: 0.02 Da

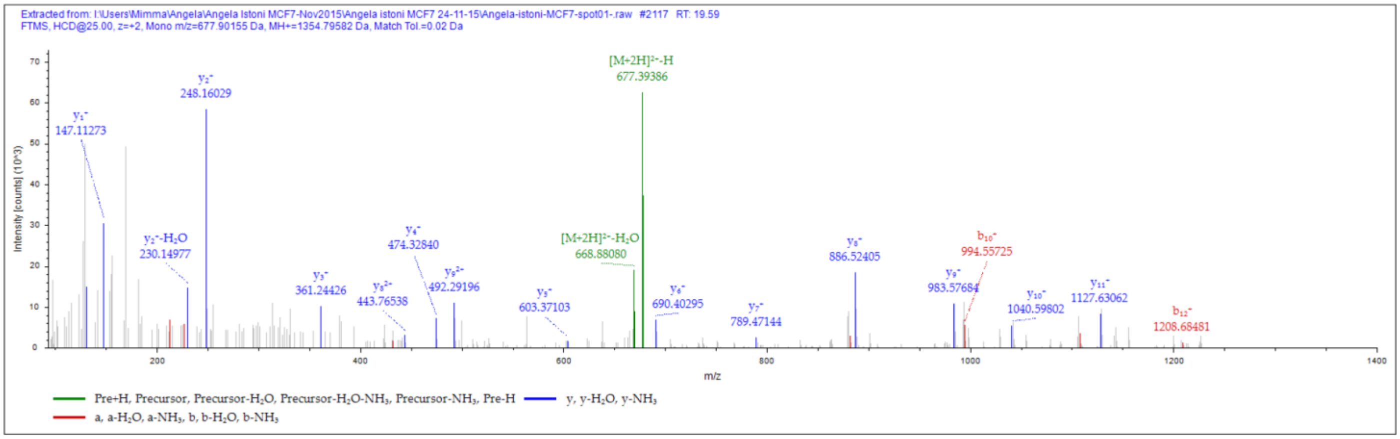


| #1 | a⁺ | a²⁺ | b⁺ | b²⁺ | Seq. | y⁺ | y²⁺ | #2 |
|---|---|---|---|---|---|---|---|---|
| 1 | 12.913.863 | 6.507.295 | 15.713.355 | 7.907.041 | K-Dimethyl | | | 13 |
| 2 | 20.017.575 | 10.059.151 | 22.817.067 | 11.458.897 | A | 119.866.784 | 59.983.756 | 12 |
| 3 | 28.720.778 | 14.410.753 | 31.520.270 | 15.810.499 | S | 112.763.072 | 56.431.900 | 11 |
| 4 | 34.422.925 | 17.261.826 | 37.222.417 | 18.661.572 | G | 104.059.869 | 52.080.298 | 10 |
| 5 | 44.128.202 | 22.114.465 | 46.927.694 | 23.514.211 | P | 98.357.722 | 49.229.225 | 9 |
| 6 | 53.833.479 | 26.967.103 | 56.632.971 | 28.366.849 | P | 88.652.445 | 44.376.586 | 8 |
| 7 | 63.740.321 | 31.920.524 | 66.539.813 | 33.320.270 | V | 78.947.168 | 39.523.948 | 7 |
| 8 | 72.443.524 | 36.272.126 | 75.243.016 | 37.671.872 | S | 69.040.326 | 34.570.527 | 6 |
| 9 | 85.347.784 | 42.724.256 | 88.147.276 | 44.124.002 | E | 60.337.123 | 30.218.925 | 5 |
| 10 | 96.656.191 | 48.378.459 | 99.455.683 | 49.778.205 | L | 47.432.863 | 23.766.795 | 4 |
| 11 | 107.964.598 | 54.032.663 | 110.764.090 | 55.432.409 | I | 36.124.456 | 18.112.592 | 3 |
| 12 | 118.069.366 | 59.085.047 | 120.868.858 | 60.484.793 | T | 24.816.049 | 12.458.388 | 2 |
| 13 | | | | | K | 14.711.281 | 7.406.004 | 1 |

**ASGPPVSELITK$_{Methyl}$** **H1.2K45 – H1.3K46**

Sequence: ASGPPVSELITK, K12-Methyl (14.01565 Da)
Charge: +2, Monoisotopic m/z: 606.84552 Da (+0.14 mmu/+0.22 ppm), MH+: 1212.68376 Da, RT: 22.66 min,
Identified with: Sequest HT (v1.3); XCorr:2.25, Ions matched by search engine: 0/0
Fragment match tolerance used for search: 0.02 Da

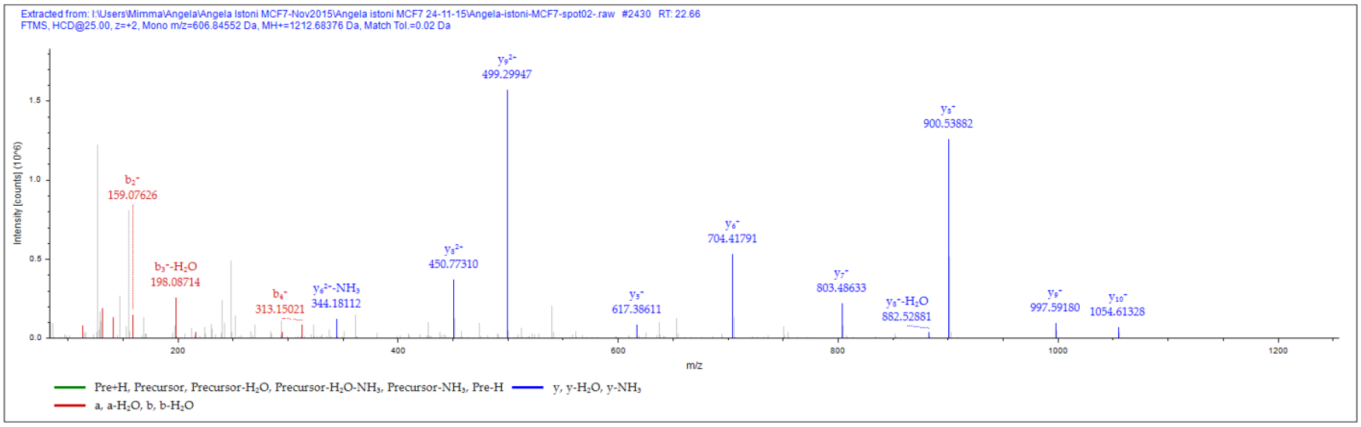


| #1 | a⁺ | a²⁺ | b⁺ | b²⁺ | Seq. | y⁺ | y²⁺ | #2 |
|---|---|---|---|---|---|---|---|---|
| 1 | 4.404.948 | 2.252.838 | 7.204.440 | 3.652.584 | A | | | 12 |
| 2 | 13.108.151 | 6.604.439 | 15.907.643 | 8.004.185 | S | 114.164.637 | 57.132.682 | 11 |
| 3 | 18.810.298 | 9.455.513 | 21.609.790 | 10.855.259 | G | 105.461.434 | 52.781.081 | 10 |
| 4 | 28.515.575 | 14.308.151 | 31.315.067 | 15.707.897 | P | 99.759.287 | 49.930.007 | 9 |
| 5 | 38.220.852 | 19.160.790 | 41.020.344 | 20.560.536 | P | 90.054.010 | 45.077.369 | 8 |
| 6 | 48.127.694 | 24.114.211 | 50.927.186 | 25.513.957 | V | 80.348.733 | 40.224.730 | 7 |
| 7 | 56.830.897 | 28.465.812 | 59.630.389 | 29.865.558 | S | 70.441.891 | 35.271.309 | 6 |
| 8 | 69.735.157 | 34.917.942 | 72.534.649 | 36.317.688 | E | 61.738.688 | 30.919.708 | 5 |
| 9 | 81.043.564 | 40.572.146 | 83.843.056 | 41.971.892 | L | 48.834.428 | 24.467.578 | 4 |
| 10 | 92.351.971 | 46.226.349 | 95.151.463 | 47.626.095 | I | 37.526.021 | 18.813.374 | 3 |
| 11 | 102.456.739 | 51.278.733 | 105.256.231 | 52.678.479 | T | 26.217.614 | 13.159.171 | 2 |
| 12 | | | | | K-Methyl | 16.112.846 | 8.106.787 | 1 |

## ALAAAGYDVEK$_{Methyl}$ H1.2K74 – H1.3K75

Sequence: ALAAAGYDVEK, K11-Methyl (14.01565 Da)
Charge: +2, Monoisotopic m/z: 561.29510 Da (-0.42 mmu/-0.75 ppm), MH+: 1121.58293 Da, RT: 18.20 min,
Identified with: Sequest HT (v1.3); XCorr:3.13, Ions matched by search engine: 0/0
Fragment match tolerance used for search: 0.02 Da

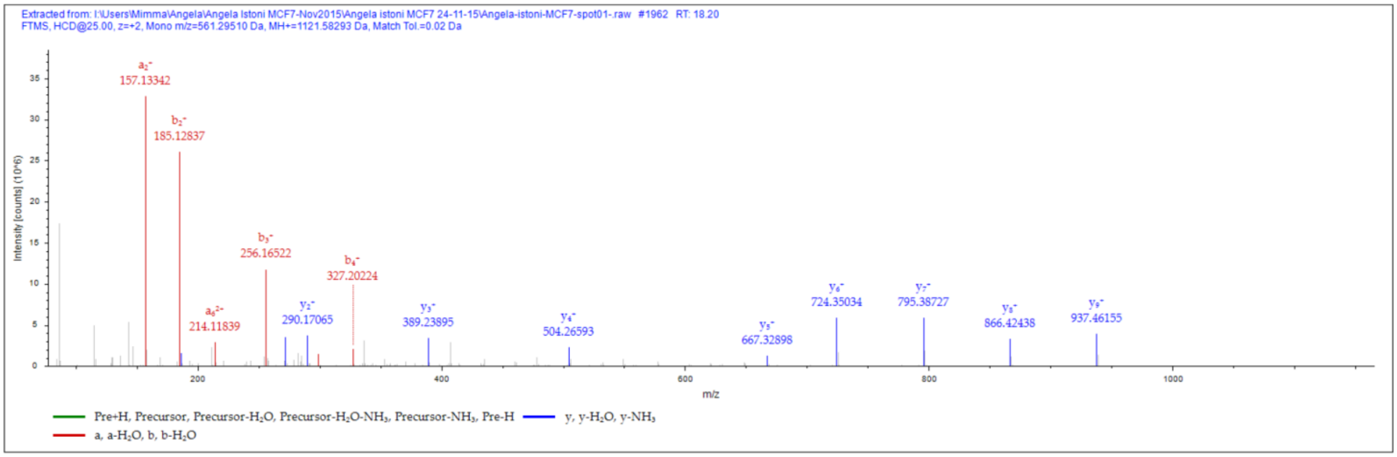


| #1 | a+ | a2+ | b+ | b2+ | Seq. | y+ | y2+ | #2 |
|---|---|---|---|---|---|---|---|---|
| 1 | 4.404.948 | 2.252.838 | 7.204.440 | 3.652.584 | A | | | 11 |
| 2 | 15.713.355 | 7.907.041 | 18.512.847 | 9.306.787 | L | 105.054.665 | 52.577.696 | 10 |
| 3 | 22.817.067 | 11.458.897 | 25.616.559 | 12.858.643 | A | 93.746.258 | 46.923.493 | 9 |
| 4 | 29.920.779 | 15.010.753 | 32.720.271 | 16.410.499 | A | 86.642.546 | 43.371.637 | 8 |
| 5 | 37.024.491 | 18.562.609 | 39.823.983 | 19.962.355 | A | 79.538.834 | 39.819.781 | 7 |
| 6 | 42.726.638 | 21.413.683 | 45.526.130 | 22.813.429 | G | 72.435.122 | 36.267.925 | 6 |
| 7 | 59.032.970 | 29.566.849 | 61.832.462 | 30.966.595 | Y | 66.732.975 | 33.416.851 | 5 |
| 8 | 70.535.665 | 35.318.196 | 73.335.157 | 36.717.942 | D | 50.426.643 | 25.263.685 | 4 |
| 9 | 80.442.507 | 40.271.617 | 83.241.999 | 41.671.363 | V | 38.923.948 | 19.512.338 | 3 |
| 10 | 93.346.767 | 46.723.747 | 96.146.259 | 48.123.493 | E | 29.017.106 | 14.558.917 | 2 |
| 11 | | | | | K-Methyl | 16.112.846 | 8.106.787 | 1 |

**ALAAAGYDVEK$_{Dimethyl}$** **H1.2K74 – H1.3K75**

Sequence: ALAAAGYDVEK, K11-Dimethyl (28.03130 Da)
Charge: +2, Monoisotopic m/z: 568.30347 Da (+0.12 mmu/+0.21 ppm), MH+: 1135.59966 Da, RT: 20.27 min,
Identified with: Sequest HT (v1.3); XCorr:2.87, Ions matched by search engine: 0/0
Fragment match tolerance used for search: 0.02 Da

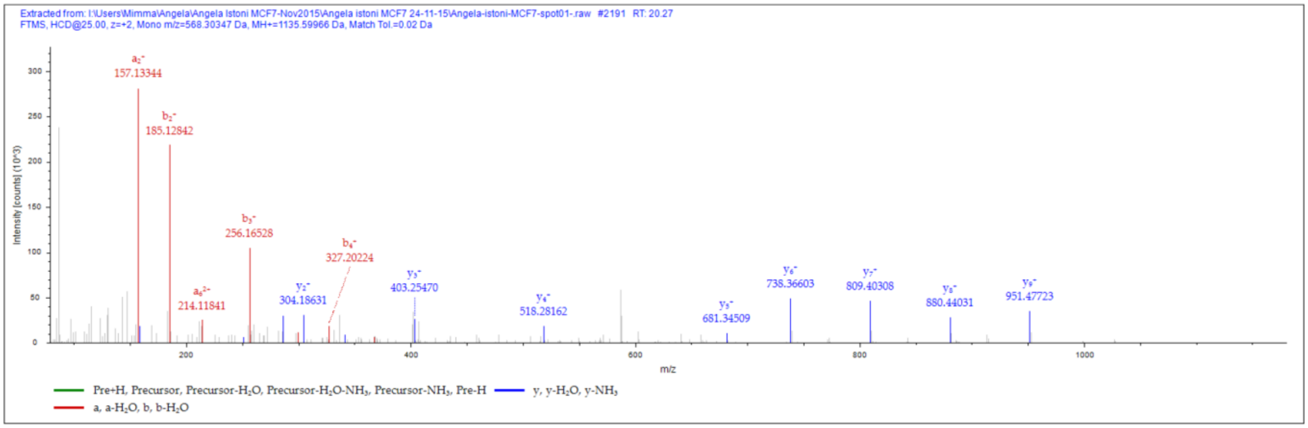


| #1 | a⁺ | a²⁺ | b⁺ | b²⁺ | Seq. | y⁺ | y²⁺ | #2 |
|---|---|---|---|---|---|---|---|---|
| 1 | 4.404.948 | 2.252.838 | 7.204.440 | 3.652.584 | A | | | 11 |
| 2 | 15.713.355 | 7.907.041 | 18.512.847 | 9.306.787 | L | 106.456.230 | 53.278.479 | 10 |
| 3 | 22.817.067 | 11.458.897 | 25.616.559 | 12.858.643 | A | 95.147.823 | 47.624.275 | 9 |
| 4 | 29.920.779 | 15.010.753 | 32.720.271 | 16.410.499 | A | 88.044.111 | 44.072.419 | 8 |
| 5 | 37.024.491 | 18.562.609 | 39.823.983 | 19.962.355 | A | 80.940.399 | 40.520.563 | 7 |
| 6 | 42.726.638 | 21.413.683 | 45.526.130 | 22.813.429 | G | 73.836.687 | 36.968.707 | 6 |
| 7 | 59.032.970 | 29.566.849 | 61.832.462 | 30.966.595 | Y | 68.134.540 | 34.117.634 | 5 |
| 8 | 70.535.665 | 35.318.196 | 73.335.157 | 36.717.942 | D | 51.828.208 | 25.964.468 | 4 |
| 9 | 80.442.507 | 40.271.617 | 83.241.999 | 41.671.363 | V | 40.325.513 | 20.213.120 | 3 |
| 10 | 93.346.767 | 46.723.747 | 96.146.259 | 48.123.493 | E | 30.418.671 | 15.259.699 | 2 |
| 11 | | | | | K-Dimethyl | 17.514.411 | 8.807.569 | 1 |

**ALAAAGYDVEK$_{\text{Trimethyl}}$** **H1.3K75**

Sequence: ALAAAGYDVEK, K11-Trimethyl (42.04695 Da)
Charge: +2, Monoisotopic m/z: 575.31073 Da (-0.44 mmu/-0.77 ppm), MH+: 1149.61418 Da, RT: 22.39 min,
Identified with: Sequest HT (v1.3); XCorr:2.95, Ions matched by search engine: 0/0
Fragment match tolerance used for search: 0.02 Da

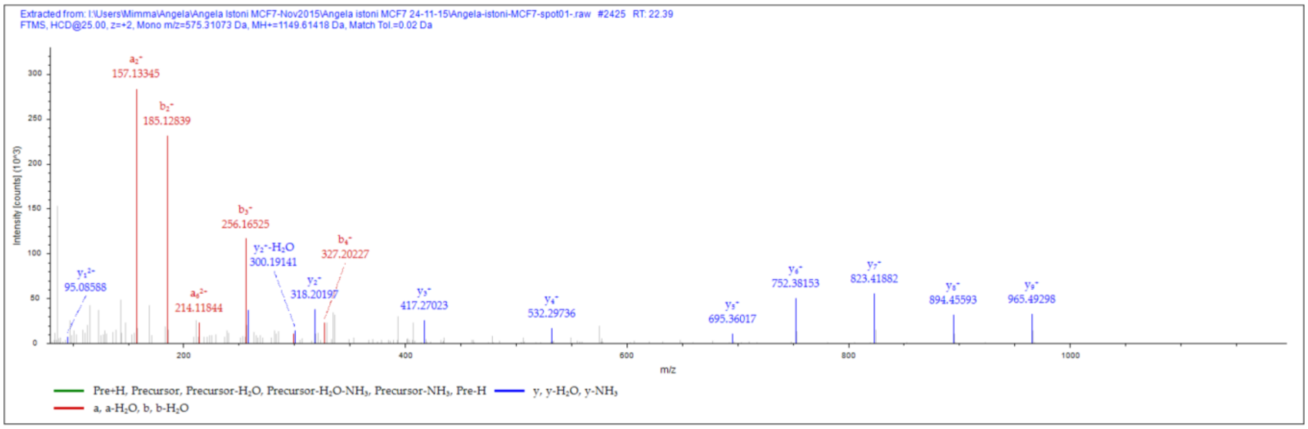


| #1 | a⁺ | a²⁺ | b⁺ | b²⁺ | Seq. | y⁺ | y²⁺ | #2 |
|---|---|---|---|---|---|---|---|---|
| 1 | 4.404.948 | 2.252.838 | 7.204.440 | 3.652.584 | A | | | 11 |
| 2 | 15.713.355 | 7.907.041 | 18.512.847 | 9.306.787 | L | 107.857.795 | 53.979.261 | 10 |
| 3 | 22.817.067 | 11.458.897 | 25.616.559 | 12.858.643 | A | 96.549.388 | 48.325.058 | 9 |
| 4 | 29.920.779 | 15.010.753 | 32.720.271 | 16.410.499 | A | 89.445.676 | 44.773.202 | 8 |
| 5 | 37.024.491 | 18.562.609 | 39.823.983 | 19.962.355 | A | 82.341.964 | 41.221.346 | 7 |
| 6 | 42.726.638 | 21.413.683 | 45.526.130 | 22.813.429 | G | 75.238.252 | 37.669.490 | 6 |
| 7 | 59.032.970 | 29.566.849 | 61.832.462 | 30.966.595 | Y | 69.536.105 | 34.818.416 | 5 |
| 8 | 70.535.665 | 35.318.196 | 73.335.157 | 36.717.942 | D | 53.229.773 | 26.665.250 | 4 |
| 9 | 80.442.507 | 40.271.617 | 83.241.999 | 41.671.363 | V | 41.727.078 | 20.913.903 | 3 |
| 10 | 93.346.767 | 46.723.747 | 96.146.259 | 48.123.493 | E | 31.820.236 | 15.960.482 | 2 |
| 11 | | | | | K-Trimethyl | 18.915.976 | 9.508.352 | 1 |

## SLVSK$_{Acetyl}$GTLVQTK H1.2K89 – H1.3K90

Sequence: SLVSKGTLVQTK, K5-Acetyl (42.01057 Da)
Charge: +2, Monoisotopic m/z: 651.88495 Da (-0.09 mmu/-0.13 ppm), MH+: 1302.76262 Da, RT: 20.42 min,
Identified with: Sequest HT (v1.3); XCorr:3.03, Ions matched by search engine: 0/0
Fragment match tolerance used for search: 0.02 Da

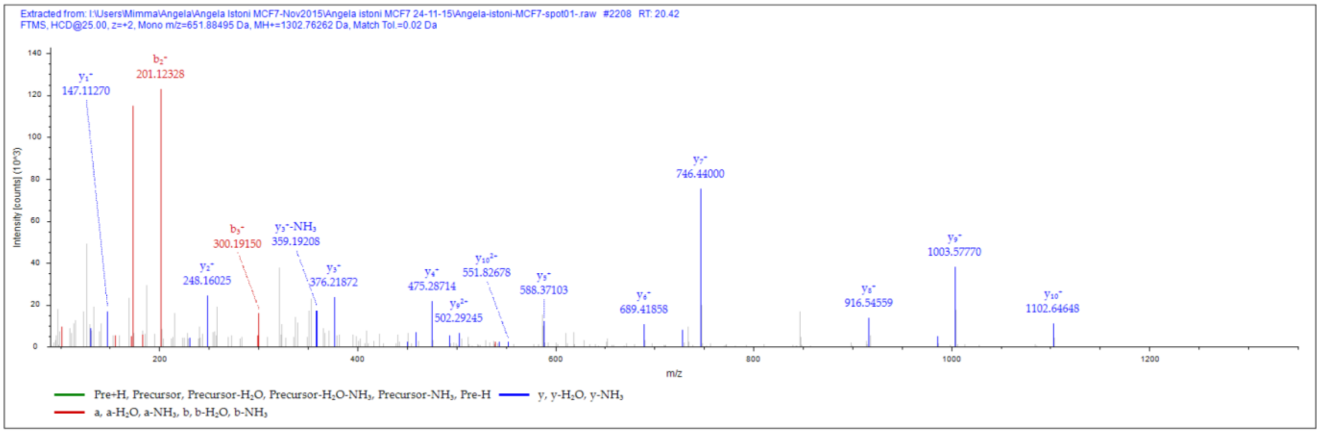


| #1 | a⁺ | a²⁺ | b⁺ | b²⁺ | Seq. | y⁺ | y²⁺ | #2 |
|---|---|---|---|---|---|---|---|---|
| 1 | 6.004.439 | 3.052.583 | 8.803.931 | 4.452.329 | S | | | 12 |
| 2 | 17.312.846 | 8.706.787 | 20.112.338 | 10.106.533 | L | 121.573.077 | 60.836.902 | 11 |
| 3 | 27.219.688 | 13.660.208 | 30.019.180 | 15.059.954 | V | 110.264.670 | 55.182.699 | 10 |
| 4 | 35.922.891 | 18.011.809 | 38.722.383 | 19.411.555 | S | 100.357.828 | 50.229.278 | 9 |
| 5 | 52.933.445 | 26.517.086 | 55.732.936 | 27.916.832 | K-Acetyl | 91.654.625 | 45.877.676 | 8 |
| 6 | 58.635.592 | 29.368.160 | 61.435.083 | 30.767.905 | G | 74.644.071 | 37.372.399 | 7 |
| 7 | 68.740.360 | 34.420.544 | 71.539.851 | 35.820.289 | T | 68.941.924 | 34.521.326 | 6 |
| 8 | 80.048.767 | 40.074.747 | 82.848.258 | 41.474.493 | L | 58.837.156 | 29.468.942 | 5 |
| 9 | 89.955.609 | 45.028.168 | 92.755.100 | 46.427.914 | V | 47.528.749 | 23.814.738 | 4 |
| 10 | 102.761.467 | 51.431.097 | 105.560.958 | 52.830.843 | Q | 37.621.907 | 18.861.317 | 3 |
| 11 | 112.866.235 | 56.483.481 | 115.665.726 | 57.883.227 | T | 24.816.049 | 12.458.388 | 2 |
| 12 | | | | | K | 14.711.281 | 7.406.004 | 1 |

## AS$_{Phospho}$GPPVSELITK H1.2S35 – H1.3S36

Sequence: ASGPPVSELITK, S2-Phospho (79.96633 Da)
Charge: +2, Monoisotopic m/z: 639.81567 Da (-5.05 mmu/-7.9 ppm), MH+: 1278.62407 Da, RT: 24.58 min,
Identified with: Sequest HT (v1.3); XCorr:2.86, Ions matched by search engine: 0/0
Fragment match tolerance used for search: 0.02 Da

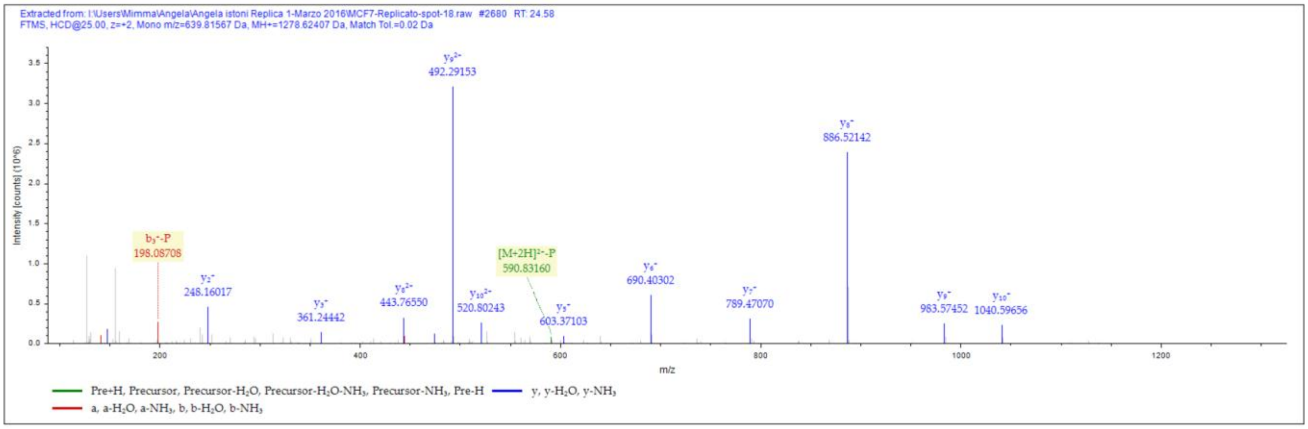


| #1 | a+ | a2+ | b+ | b2+ | Seq. | y+ | y2+ | #2 |
|---|---|---|---|---|---|---|---|---|
| 1 | 4.404.948 | 2.252.838 | 7.204.440 | 3.652.584 | A | | | 12 |
| 2 | 21.104.784 | 10.602.756 | 23.904.276 | 12.002.502 | S-Phospho | 120.759.705 | 60.430.216 | 11 |
| 3 | 26.806.931 | 13.453.829 | 29.606.423 | 14.853.575 | G | 104.059.869 | 52.080.298 | 10 |
| 4 | 36.512.208 | 18.306.468 | 39.311.700 | 19.706.214 | P | 98.357.722 | 49.229.225 | 9 |
| 5 | 46.217.485 | 23.159.106 | 49.016.977 | 24.558.852 | P | 88.652.445 | 44.376.586 | 8 |
| 6 | 56.124.327 | 28.112.527 | 58.923.819 | 29.512.273 | V | 78.947.168 | 39.523.948 | 7 |
| 7 | 64.827.530 | 32.464.129 | 67.627.022 | 33.863.875 | S | 69.040.326 | 34.570.527 | 6 |
| 8 | 77.731.790 | 38.916.259 | 80.531.282 | 40.316.005 | E | 60.337.123 | 30.218.925 | 5 |
| 9 | 89.040.197 | 44.570.462 | 91.839.689 | 45.970.208 | L | 47.432.863 | 23.766.795 | 4 |
| 10 | 100.348.604 | 50.224.666 | 103.148.096 | 51.624.412 | I | 36.124.456 | 18.112.592 | 3 |
| 11 | 110.453.372 | 55.277.050 | 113.252.864 | 56.676.796 | T | 24.816.049 | 12.458.388 | 2 |
| 12 | | | | | K | 14.711.281 | 7.406.004 | 1 |

## ALAAAGY$_{Phospho}$DVEKNNSR H1.2Y70 – H1.3Y71

Sequence: ALAAAGYDVEKNNSR, Y7-Phospho (79.96633 Da)
Charge: +2, Monoisotopic m/z: 829.87280 Da (-7.57 mmu/-9.12 ppm), MH+: 1658.73833 Da, RT: 16.31 min,
Identified with: Sequest HT (v1.3); XCorr:2.90, Ions matched by search engine: 0/0
Fragment match tolerance used for search: 0.02 Da

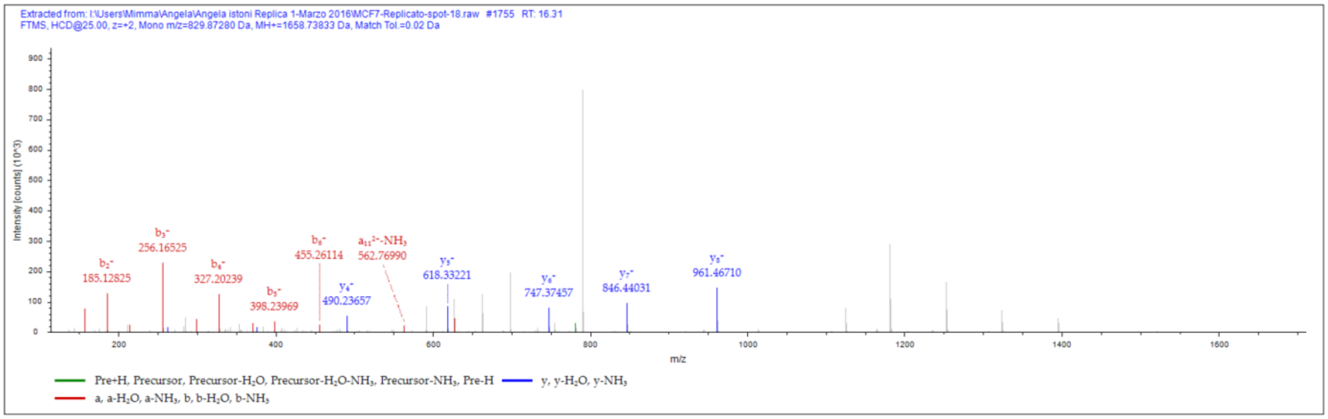


| #1 | a+ | a2+ | b+ | b2+ | Seq. | y+ | y2+ | #2 |
|---|---|---|---|---|---|---|---|---|
| 1 | 44.04948 | 22.52838 | 72.04440 | 36.52584 | A | | | 15 |
| 2 | 157.13355 | 79.07041 | 185.12847 | 93.06787 | L | 1587.71634 | 794.36181 | 14 |
| 3 | 228.17067 | 114.58897 | 256.16559 | 128.58643 | A | 1474.63227 | 737.81977 | 13 |
| 4 | 299.20779 | 150.10753 | 327.20271 | 164.10499 | A | 1403.59515 | 702.30121 | 12 |
| 5 | 370.24491 | 185.62609 | 398.23983 | 199.62355 | A | 1332.55803 | 666.78265 | 11 |
| 6 | 427.26638 | 214.13683 | 455.26130 | 228.13429 | G | 1261.52091 | 631.26409 | 10 |
| 7 | 670.29603 | 335.65165 | 698.29095 | 349.64911 | Y-Phospho | 1204.49944 | 602.75336 | 9 |
| 8 | 785.32298 | 393.16513 | 813.31790 | 407.16259 | D | 961.46979 | 481.23853 | 8 |
| 9 | 884.39140 | 442.69934 | 912.38632 | 456.69680 | V | 846.44284 | 423.72506 | 7 |
| 10 | 1013.43400 | 507.22064 | 1041.42892 | 521.21810 | E | 747.37442 | 374.19085 | 6 |
| 11 | 1141.52897 | 571.26812 | 1169.52389 | 585.26558 | K | 618.33182 | 309.66955 | 5 |
| 12 | 1255.57190 | 628.28959 | 1283.56682 | 642.28705 | N | 490.23685 | 245.62206 | 4 |
| 13 | 1369.61483 | 685.31105 | 1397.60975 | 699.30851 | N | 376.19392 | 188.60060 | 3 |
| 14 | 1456.64686 | 728.82707 | 1484.64178 | 742.82453 | S | 262.15099 | 131.57913 | 2 |
| 15 | | | | | R | 175.11896 | 88.06312 | 1 |

**Supplementary Figure 3. Fragmentation MS/MS spectra of the modified peptide carrying identified PTMs.** MS/MS data are referred to all analyzed histones. Data were analyzed by Proteome Discoverer 1.4 software. MS/MS data were searched on the Human UniProt database. For each PTM we reported: Sequence, type of modification, Charge, m/z, and RT.
